# Windows into Planetary Science: A Review of Advances in Raman Spectroscopy, Laser-Induced Breakdown Spectroscopy, and Photoluminescence Spectroscopy for Remote Sensing Applications

**DOI:** 10.1021/acsaom.6c00053

**Published:** 2026-06-10

**Authors:** Moulika Hazra, Riccardo Corpino, Pier C. Ricci

**Affiliations:** Department of Physics, 3111 University of Cagliari, Complesso Universitario di Monserrato, S.P. Monserrato-Sestu Km 0,700, 09042 Monserrato, Cagliari, Italy

**Keywords:** Remote sensing, planetary exploration, Raman spectroscopy, LIBS, optical spectroscopy, photoluminescence spectroscopy, multimodal spectroscopy

## Abstract

Laser-based optical spectroscopic techniques, namely Raman spectroscopy, laser-induced breakdown spectroscopy (LIBS), and photoluminescence (PL), are advanced analytical tools reliably used for remote sensing in space exploration and its parallels in Earth-based environments. Each of these techniques offers unique advantages for detecting and identifying minerals and other inorganic components of soil, sources of hydration, organic compounds and biosignature materials, and atmospheric gases, which together provide essential clues about planetary evolution, resource potential, and the possibility of human habitation. This Review concentrates on the advancements reported in the past 5 years in the implementation of these laser-based optical techniques for space-exploration-related studies or the equivalent. By integrating the strengths of Raman, LIBS, and PL spectroscopies within a single framework, this Review provides a unified roadmap for advancing the role of optical spectroscopy in planetary exploration.

## Graphical abstract

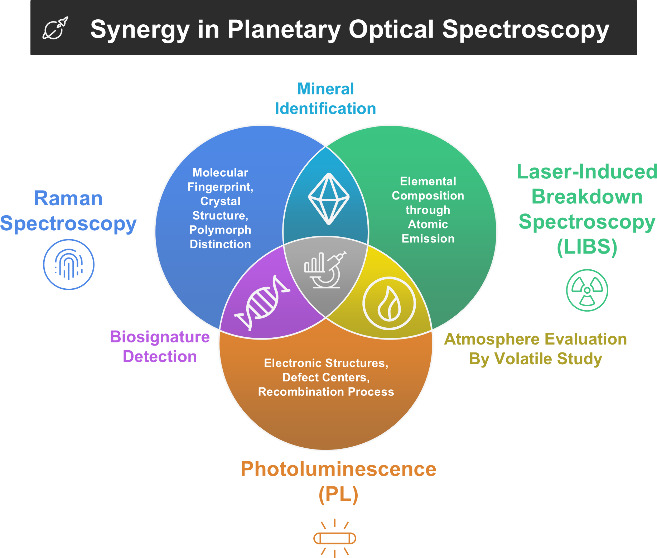

## 1 Introduction

Over the past few decades, the demand for precise, in situ characterization of planetary surfaces, atmospheres, and potential resources has intensified as space-exploration missions have shifted from simple imaging to comprehensive chemical and mineralogical analyses.^1–4^ Planetary remote sensing missions provide invaluable insights into Solar System formation and evolution, planetary processes, and the potential for life beyond Earth.^5,6^ Planetary exploration emerged with early flyby and orbiter missions like Mariner 4 (1964),^7^ Pioneer Venus Orbiter (1978),^8^ and Viking 1 Orbiter (1975),^9^ which provided the first direct imaging and atmospheric measurements of planetary bodies, laying the foundation for modern planetary observation techniques. By 2020, major achievements included global mineral mapping of Mars and Venus by Mars Express (2003)^10^ and Magellan (1989), detection of water/ice by Mars Odyssey (2001),^11,12^ atmospheric escape processes characterized by MAVEN (2013),^13^ and in situ geochemical analysis via rover-based instruments such as Curiosity (Mars Science Laboratory, 2011).^14^ These missions had provided a baseline knowledge about the atmospheres and regolith of extraterrestrial bodies by the current decade (2020s). Among the wide array of analytical approaches such as mass spectrometry,^15^ X-ray diffraction (XRD),^14^ gamma ray spectroscopy,^10^ infrared (absorption) spectroscopy (IR),^16^ gas chromatography,^17^ and reflectance spectroscopy,^18^ optical spectroscopic techniques have emerged as indispensable tools due to noncontact, high-resolution, and real-time detection of materials through light–matter interactions.^19,20^ These optical techniques not only provide elemental and molecular fingerprints, but also insights into the physical states of planetary materials, which are critical for interpreting geological history, assessing bioforms, and conceptualizing future human exploration missions.^21–23^ Hence, the past and present space missions present a high dependency on optical spectroscopic tools for in situ analysis and resource identification. This interest in the research area of “optical spectroscopy in planetary exploration” is evidenced by the number of research articles published and cited throughout the past 35 years corresponding to this field, depicted in Figure 1. The graph shows a surge in the literature in the past decade which led up to ∼10,000 citations on this area of research in the year 2025 alone. Each publication has mentioned at least one of following—Raman spectroscopy, laser-induced breakdown spectroscopy (LIBS), or photoluminescence (PL) spectroscopy—for the application in a space exploration mission or equivalent.

**Figure 1:**
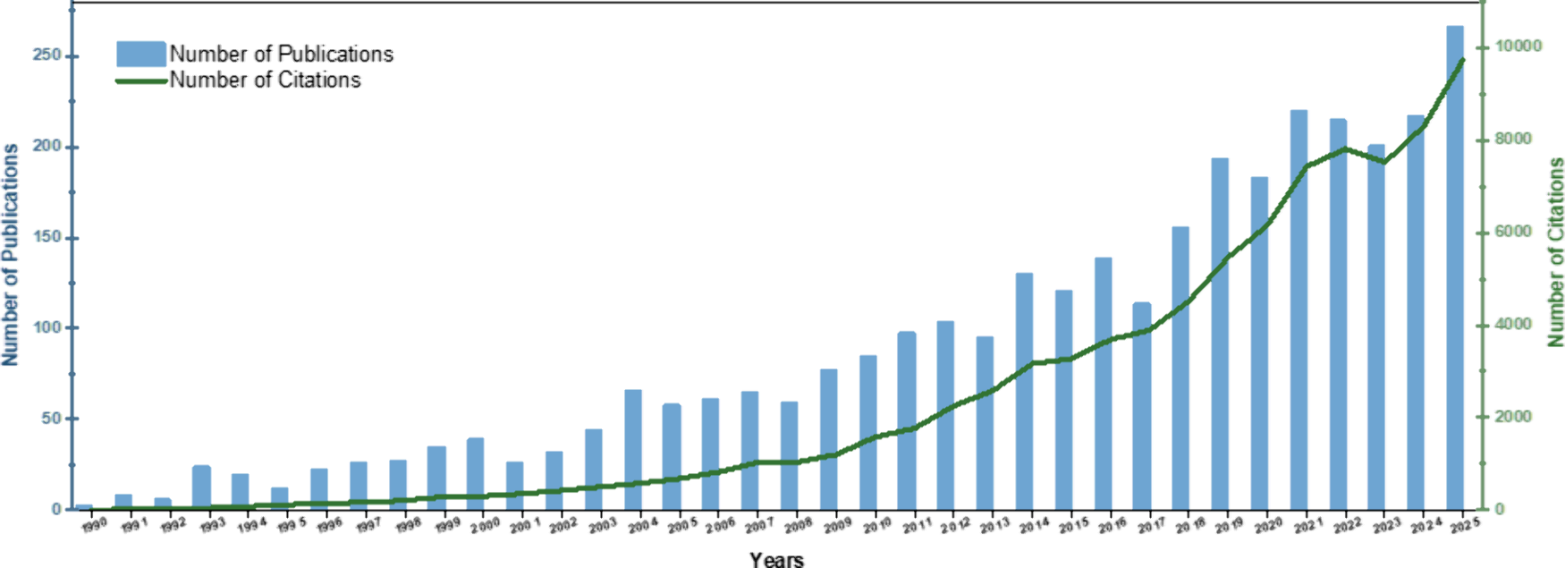
Publication and citation trends over the past 35 years for studies employing laser-based optical spectroscopic techniques (Raman, LIBS, PL, LIF) in planetary exploration. Data was retrieved from the Web of Science Core Collection using the Advanced search query through topic-based keyword searches (accessed January 30, 2026).

This statistic signifies the current need and interest in advancing the available laser based spectroscopic techniques of Raman, LIBS, and PL furthermore, even though they were advanced enough to have already been utilized in successful planetary exploration missions like NASA Mars 2020.^24^ Upcoming planetary exploration missions represent a shift toward high-precision, multi-instrument, and international deep-space exploration, with significant achievements in surface science, atmospheric studies, and astrobiology.^25–27^ Therefore, a specialized development of the spectroscopic techniques of Raman, LIBS, and PL is needed for the detection of organic/inorganic compounds, water/ice, and atmospheric constituents.

Raman spectroscopy is a powerful analytical technique that exploits the inelastic scattering of monochromatic light, typically from a laser, to provide a detailed information about molecular vibrations within a sample. It is a nondestructive technique providing information on the structure, symmetry, electronic environment, and bonding of molecules, thus allowing both quantitative and qualitative analysis of the target material.^28–31^ Its ability to acquire data in a short duration from a distance with minimum sample preparation and the versatility in identifying a wide range of organic and inorganic compounds, makes it the most sought-after optical technique for remote sensing.^32^ Raman spectroscopy is being enthusiastically utilized in space missions, like in NASA’s MARS2020 mission’s Perseverance rover with SuperCam and SHERLOC, the ESA/NASA ExoMars 2028 mission’s Raman Laser Spectrometer (RLS) included in the analytical laboratory drawer of the Rosalind Franklin rover, JAXA’s Martian Moon eXploration (MMX) mission with the IDEFIX rover, etc.^24,33–36^ This technique provides molecular-level fingerprints through vibrational transitions, enabling the identification of minerals, hydrated phases, and carbonaceous matter both in terrestrial analogue sites and on planetary missions.^37–39^

Laser-induced breakdown spectroscopy (LIBS), on the other hand, analyzes the emission spectra from a plasma, spark, electric arc, or flame spectra generated by focused laser pulses on the sample of study. This atomic emission spectrum is beneficial for elemental analysis in all forms—solid, liquid, and gas.^40–42^ This technique yields rapid data and provides in situ identification of elements without specific sample preparation techniques.^43,44^ The biggest advantage of LIBS for planetary exploration applications is its efficiency in detecting the lightest elements (H, He, Li, Be, B, C) in even low abundance levels of μg, along with multielemental composition mapping at high spatial resolution.^45,46^ Existent space exploration missions have employed LIBS for its versatility and advantages, e.g., the Martian missions’ LIBS instruments (ChemCam on the Curiosity rover, MarSCoDe on the Zhurong rover, SuperCam on the Perseverance rover, etc.).^45,47–50^

Photoluminescence (PL) spectroscopy works on the principle of the absorption of photons by a material leading to an excited state, with subsequent emission of energy as electrons migrate to lower energy states, which makes it a potent analytical technique for examining electronic transitions in materials.^51^ PL can identify optical band gaps, defect states, impurities, luminescent centers, and radiation-induced alterations in minerals, semiconductors, glasses, and organic substances, all of which bear significance in the context of planetary surfaces.^52^ Time-resolved PL (TRPL) can provide information on the lifetimes of emissions, resulting in critical insights into the processes of carrier recombination, defect trapping strategies, crystallinity levels, and the record of radiation exposure.^53,54^ Using a monochromatic, intense laser excitation for PL, enables high sensitivity and selectivity with excellent signal-to-noise ratios especially useful for spotting trace substances and organic pigments.^55^ Simply put, PL spectroscopy and its derivatives are exceptionally well-suited for planetary research as they function effectively in extreme environments, and improve the identification of nuanced compositional and structural markers pertinent to planetary evolution and astrobiology.^54,57,58^ The Europan Molecular Indicators of Life Investigation (EMILI) will employ PL-based techniques for searching biosignatures on Jupiter’s moon with the Europa lander mission.^59^

Figure 2 shows a basic comparative summary of Raman, LIBS, and PL spectroscopies based on their principle of operation, target sample/application, and advantages/disadvantages. These individual techniques have demonstrated remarkable capabilities for extraterrestrial studies. In the context of extraterrestrial missions, where payload capacity and instrument redundancy are severely limited, it becomes essential both to use one technique for multiple analytical targets and to employ multiple techniques for cross-verification of the same element or molecule, ensuring confidence in results under extreme and variable conditions. As humanity envisions future commercialization and colonization of Mars and the moon, understanding the mineralogical, geochemical, and atmospheric environments of these celestial bodies becomes crucial. The growing global interest in planetary exploration driven by the quest to identify traces of water, organic molecules, and potential biosignatures demands an integrative perspective.

**Figure 2:**
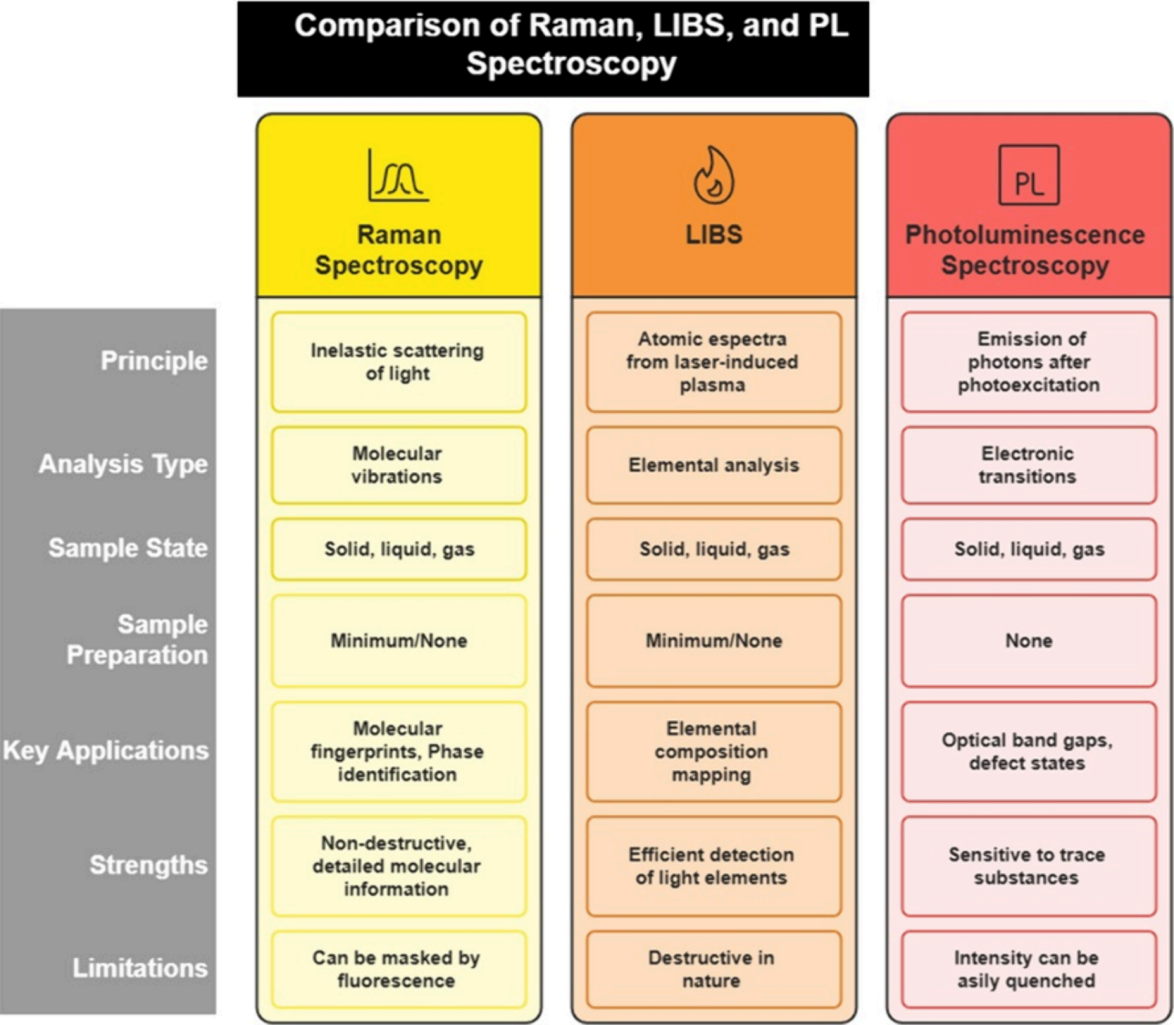
Comparative summary of Raman, LIBS, and PL spectroscopy.

Existing review articles on spectroscopic techniques for planetary exploration are generally targeted toward a particular spectroscopic technique or application. Table 1 summarizes some of the recent review articles which focus on the prospectus of “optical spectroscopy for planetary exploration”. Most of the reported reviews focus on Raman or LIBS,^32,40,63^ without a discussion on PL, while those that cover multiple methods are often heavily instrumentation-oriented^34,70–72^ rather than application-driven. A significant number of publications discuss data refinement and modeling approaches for Raman and LIBS; however, these tend to prioritize analytical workflows over their actual scientific applications.^73–76^

**Table 1:** Recently Published Review Articles with a Focus on Planetary Exploration

**Author, Journal, Year**	**Key Focus of the Review**	**Identified Gaps (Addressed in This Review)**	**Ref**
Ntziouni et al., *Applied Spectroscopy*, 2022	List, categorize, and undertake an exhaustive examination of the various benchmarks, protocols, and methodologies associated with Raman spectroscopy	Other optical technologies not discussed	^60^
Michalski et al., *Nature Astronomy*, 2022	Discussion of the geochemical and biosignatures reported from the lakes on Mars	No discussion of techniques used	^61^
Jia et al., *Space: Science & Technology*, 2023	Techniques applied for remote detection or analysis of biosignatures; Raman and LIBS mentioned	Discussion of only one application	^62^
Thomas et al., *Applied Spectroscopy Reviews*, 2024	Complete discussion on LIBS methodologies and types of LIBS; instrumentation focused	Other optical technologies not discussed	^40^
Ferreira et al., *JAAS*, 2024	Critical aspects and emerging trends in LIBS, focusing on calibration challenges, integrating complementary techniques (data fusion), and applying data science for spectral analysis	Other optical technologies not discussed	^42^
Saeidfirozeh et al., *TrAC*, 2024	Prospect of LIBS in remote sensing for planetary explorations	Other optical technologies not discussed	^63^
Sun et al., *JAAS*, 2024	LIBS technology in the field of atmospheric particulate matter detection	Other optical technologies not discussed	^64^
		Discussion of only one application	
Yao et al., *TrAC*, 2024	Progress in multitechnique-integrated LIBS for remote sensing applications	Only technique oriented, no discussion on target materials	^65^
Yu et al., *Remote Sensing*, 2025	Evolution of Mars water–ice detection technology from 1990 to 2024	No discussion of optical spectroscopy	^66^
Yan et al., *JAAS*, 2025	Qualitative and quantitative advancements in rock detection through LIBS	Other optical technologies not discussed	^67^
Wiens et al., *Minerals*, 2025	Mars exploration with LIBS in ChemCam, SuperCam and MarSCoDe instruments	Other optical technologies not discussed	^68^
Lichtenberg et al., *Science*, 2025	Understanding the interior of exoplanets by observing their atmosphere	Discussion of only one application	^69^

The literature survey depicted that Raman spectroscopy dominated such discussions on planetary exploration, followed by LIBS, and PL spectroscopy lagged. Reviews that adopt an application-oriented approach, such as those addressing mineral detection, soil composition, atmospheric study, organic matter detection, or water identification, typically emphasize only one specific utilization and do not report more than one target material.^77–80^ Many studies, such as that of Rull et al., combine optical spectroscopy with other characterization tools based on entirely different physical principles, diluting the focus on spectroscopic methodologies.^32^ While certain reports concentrate solely on laser-based spectroscopies for planetary exploration, including the works of Wien et al. and Raha et al., they remain primarily devoted to the description of instruments and mission payloads, with little discussion on the target samples or specific scientific objectives these techniques aim to address.^68,81,82^ There is, therefore, a clear need for a comprehensive, application-focused review that bridges the gap between instrumentation and scientific objectives: one that also brings photoluminescence spectroscopy to the main light along with Raman spectroscopy and laser-induced breakdown spectroscopy (LIBS) and provides discussion on their synergistic employment to study diverse extraterrestrial materials, including soils, minerals, water, and atmospheric gases.

This integrated understanding of how the complementary methods of Raman, LIBS, and PL can jointly enhance detection accuracy and broaden the scope of scientific inquiry is addressed in this Review. Figure 3 depicts this idea in a graphical way.

**Figure 3:**
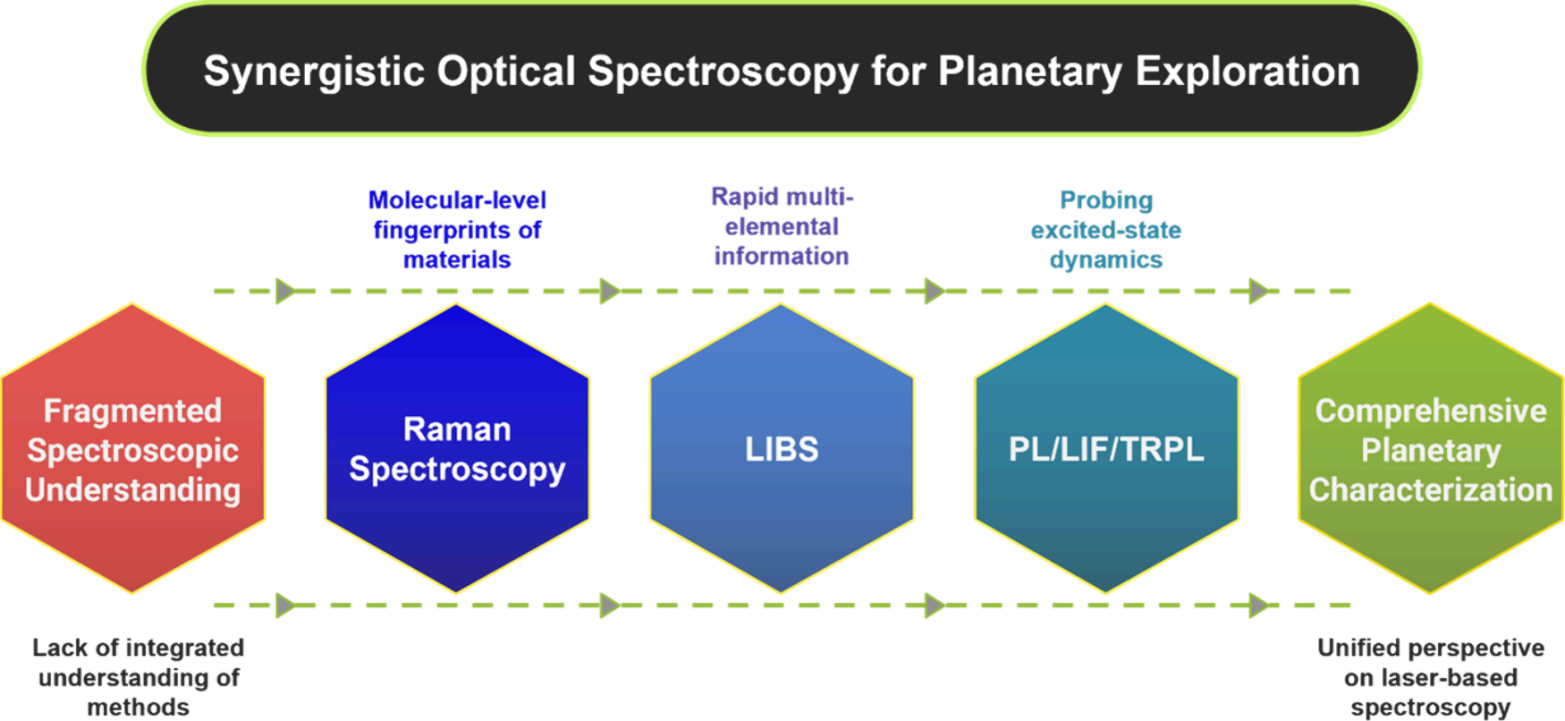
Schematic representing the importance of multimodal optical spectroscopic techniques for planetary explorations.

A detailed overview of the use and advancements of optical spectroscopic techniques of Raman/LIBS/PL for a range of material detection reported in the past 5 years (2021–2025) is provided here, focusing subsections on inorganics and minerals in soil, water/hydrates, organics and biomolecules, and atmospheric gases. The focus of this article lies in providing a guide to the material scientists working in this area to be able to utilize these spectroscopic techniques more efficiently for their target detection (apart from data treatment procedures) and providing spectroscopists a summary of the recent and possible developments. This Review can serve as a critical reference for the material science and spectroscopy community, highlighting specific material detection, existing gaps, technological bottlenecks, and unexplored opportunities in the optimal utilization of Raman, LIBS, and PL techniques for planetary and astrobiological research. The primary sections 2–5) report the recent five-year advancements, which are analyzed and summarized in the subsequent sections 6,7), with a discussion of challenges and mitigation techniques—instrumental, technical, and data interpretative. The existing advantages and limitations of each of the three spectroscopic techniques are summarized, and the importance of the combined use of these techniques is discussed.

## 2 Exploring Inorganics in Extraterrestrial Soils

The soil particles found on a planet are key indicators of surface processes, environmental dynamics, and weathering mechanisms operating on those bodies.^83^ Understanding the nature of planetary soil is also essential for mission planning, as it influences the thermal properties, optical reflectance, and overall instrument performance of the landed missions.^84^ One of the key components in the soil of other planets which is of high human interest is minerals.

Minerals are the building blocks of modern human civilization as they are vastly utilized to obtain metals, ceramics, semiconductors, and other functional materials for applications in jewelry, electronics, construction, energy storage, and catalysis.^85,86^ The existence of minerals on the surface of a planet indicates a history of geochemical processes like a volcanic activity, which can reveal the planet’s thermal evolution, crustal differentiation, and potential for hosting resources. Minerals are primarily composed of oxides, silicates, carbonates, sulfates, and sulfides of metals. Detecting both metallic and nonmetallic components is essential for understanding the geochemical environment and formation mechanisms.^87,88^ Furthermore, practical applications and mining operations depend on identifying the specific mineral phase, as different polymorphs exhibit distinct properties.^89^ Therefore, multiple complementary spectroscopic techniques are required for comprehensive mineralogical characterization.

### 2.1 Raman for Mineral Phase Identification

Raman spectroscopy’s sensitivity to molecular vibrations enables identification of silicates, carbonates, sulfates, oxides, and phosphates through characteristic spectral signatures.^90^ The technique’s sensitivity to crystal structure enables identification of alteration products and weathering minerals that record past environmental conditions.^91^ Its ability to detect amorphous phases represents a significant advantage for soil analysis.^92^ Many planetary soils contain substantial amorphous components produced by impact processing, volcanic glass formation, or aqueous alteration.^93^ While X-ray diffraction struggles with amorphous materials, Raman spectroscopy can detect broad bands characteristic of disordered structures, providing insights into soil formation processes.^94^

Over the past decade, Raman spectroscopy has been successfully deployed on numerous planetary exploration missions, yielding extensive mineralogical data sets.^38,95–97^ A notable implementation is the SuperCam and SHERLOC (Scanning Habitable Environments with Raman and Luminescence for Organics and Chemicals) instruments aboard NASA’s Mars 2020 Perseverance rover, which has provided high-resolution molecular and mineralogical information from the Jezero Crater.^98^ SHERLOC has complemented SuperCam’s remote measurements with high-resolution deep-UV Raman spectroscopy at 248.6 nm. During the crater floor campaign, SHERLOC detected multiple mineral classes including sulfates, carbonates, phosphates, and silicates, with particular success in identifying fine-grained alteration phases.^98^ The instrument successfully identified primary minerals including k-feldspar, plagioclase, quartz, muscovite, and rutile, along with minor phases such as phyllosilicates, calcite, gypsum, and hematite.^99^

During the first 1000 sols of operation, SuperCam successfully identified diverse mineral assemblages including olivine, pyroxenes (both low-Ca and high-Ca varieties), plagioclase, Cr–Fe–Ti oxides, and phosphates in the Séítah formation.^100,101^ Figure 4a represents the Raman spectra of different carbonate-based minerals recorded by Veneranda and co-workers as a database for SuperCam on Mars.^102^ It discriminated between carbonate polymorphs and detected carbonates in igneous rocks on the Jezero crater floor, providing evidence for aqueous alteration processes.^102,103^ The instrument’s remote operation capability enabled analysis of undisturbed soil surfaces, avoiding potential contamination or alteration from contact instruments.^101^

**Figure 4:**
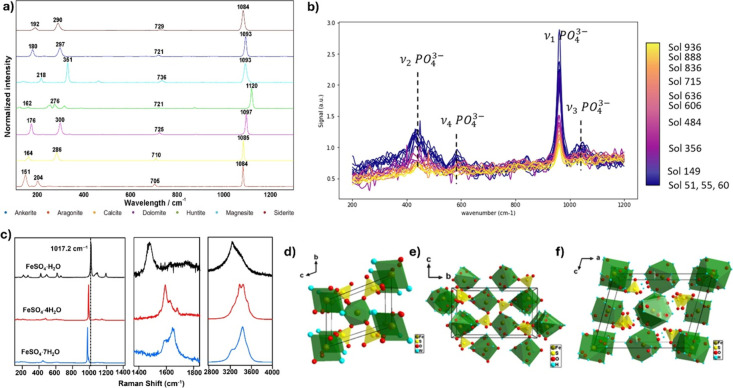
a) Representative Raman spectra obtained from the carbonate phases. (Adapted from ref 102. Copyright 2023 The Authors under a CC BY 4.0 license.) b) Mars Raman spectra of an apatite sample acquired with SuperCam on apatite SCCT (TAPAG) on different sols throughout the mission, normalized to the mean signal. The plots are color coded with sol number (*x* axis shows wavenumber in cm^–1^). (Image and caption reproduced from ref 104 under a CC BY 4.0 license.) c) Raman spectra of three hydrated ferrous sulfates. Crystal structures of d) FeSO_4_·H_2_O, e) FeSO_4_·4H_2_O, and f) FeSO_4_·7H_2_O. (Panels c–f adapted from ref 97. Copyright 2024 The Authors under a CC BY 4.0 license.)

A concern that has been brought to light by Clavé et al. is that minerals undergo an alteration in their structure on the surface of planets like Mars, where exposure to UV radiation introduces defects in the material.^104^ This is was proven by the Raman spectroscopy of a synthetic apatite sample throughout the initial 950 Martian days (sols). As shown in Figure 4b, a reduction in the relative intensity of the Raman signal ν_1_ (symmetric stretching of PO_4_^3–^) at 960 cm^–1^ in relation to the overall signal was noted with the higher duration of radiation exposure.^104^

Recently, three hydrated ferrous sulfate samples were synthesized in laboratory and analyzed through Raman spectroscopy to find any unique characteristics shown in Figure 4c (for instance, the ν_1_ and ν_3_ peaks of SO_2_^4–^ tetrahedra) which could provide fresh perspectives for distinguishing various ferrous sulfates on Mars.^97^ Figure 4d–f depicts the crystal structures of the three different ferrous sulfates, indicating the efficiency of Raman in distinguishing similar minerals with different phases.

Raman spectroscopy has also been employed to investigate the stability of sulfates present on Mars (specifically gypsum, syngenite, and görgeyite) in relation to high temperature conditions.^105^ The findings indicated a transition toward lower wavenumbers as the temperature increased for all samples, until reaching the inflection temperature, where phase transitions took place. This trend facilitated the approximation of Raman band wavenumbers at defined temperatures, in addition to the identification of the temperature at which a particular spectrum was obtained.^105^

Apart from the spectrometers already deployed in space, there are multiple research works focusing on the advancement of this technique for a higher efficiency of mineral detection. A work by Tripathi et al. showed the supremacy of Raman spectroscopy in unveiling different silica polymorphs by studying a total of seven terrestrial rock samples from the Earth and its moon, comprising both sedimentary and metamorphic types, to obtain Si–O–Si stretching.^38^ More planetary scientists are trying to develop advanced Raman systems for the study of soil compositions. Li et al. used a self-made laser Raman spectroscopy system to analyze three main components of lunar regolith, feldspar, olivine, and pyroxene, returned from the CE-5 mission.^106^ The Indian Space Research Organisation (ISRO) is engineering a Raman spectrometer for prospective lunar expeditions to analyze mineral content in lunar regolith with a resolution of 8 cm^–1^ across the wavenumber spectrum from 150 cm^–1^ to 3800 cm^–1^.^82^ The instrument adopts a monostatic design, integrating a unified optical system for laser alignment, sample placement, and signal detection to reduce misalignment errors while prioritizing mass, volume, and sensitivity due to the rigorous demands of extraterrestrial missions.^82^

The rovers and remote sensing spectrometers used for planetary missions are generally semiautonomous and further improvement in the survey and analysis of minerals is direly needed for a more effective reading to make them completely autonomous. Johnsen and co-workers proposed the use of a coregistered dual-band Raman spectrometer for autonomous mineral classification.^107^ They showed that using two excitation lasers of different wavelengths (532 and 785 nm) simultaneously to obtain the same spot-size on the same sample was the answer. They studied 191 rocks to identify the minerals pyroxene, olivine, potassium feldspar, quartz, mica, gypsum, and plagioclase, testing on a novel sample set for single-mineral classification and demonstrating accuracy scores up to 100% (varying by mineral), with a total classification rate (all minerals) of 91%.^107^

To identify practical challenges and refine analytical strategies for in situ data interpretation and sample selection for the Perseverance and ExoMars missions, Lalla et al. used a simulator of the ExoMars Raman Laser Spectrometer (RLS Sim) (designed to replicate the behavior of the instrument onboard the Rosalind Franklin rover) to analyze rocks and soils collected during the CanMars rover mission in Hanksville, Utah—a terrestrial analogue to Mars.^27^ By performing detailed Raman spectral analyses, the team assessed instrument sensitivity, data quality, and mineral detection capabilities, successfully identifying a range of compounds including oxides, sulfates, carbonates, and feldspars.^27^

A variety of terrestrial analogue sites, including Iceland (tholeiitic basalts), Scotland (ferropicrites), the Canary Islands (alkali-rich rocks), and the Granby formation in the USA (Tenerife, basaltic tuffs), as well as the Leka ophiolite complex in Norway (serpentinized peridotite), have been investigated to serve as effective analogues for studying the processes associated with Martian mantle-plume-fed volcanism, as well as the development of alkali-rich crustal units on Noachian Mars.^108^ In the Atacama Desert ExoFiT trial, Raman spectroscopy has identified phyllosilicates, sulfates, and carbonates in soil samples.^99^ The integration enabled comprehensive characterization of soil mineralogy and chemistry with implications for understanding past aqueous conditions.^99^ These reports suggest that the development of next-generation Raman instruments should emphasize an enhanced sensitivity and operational flexibility.

### 2.2 LIBS for Elemental Composition

The ability of laser-induced breakdown spectroscopy to detect major, minor, and trace elements across the periodic table makes it invaluable for geochemical characterization and petrogenetic interpretation.^109–111^ It excels at rapid elemental analysis of heterogeneous soil and dust samples, providing statistical characterization through multiple-point measurements.^112,113^ In the past decade’s space missions, utilization of LIBS and its prospects has been studied and applied, e.g., ChemCam on the Curiosity rover on Mars.^114^

SuperCam’s LIBS database includes 332 calibration standards, enabling quantitative analysis of major elements (Si, Fe, Mg, Al, Ca, Na, K, Ti) and detection of trace elements including rare earth elements.^72^ The instrument has successfully characterized geochemical variations along petrological traverses, revealing systematic changes in olivine composition (Mg) and bulk rock chemistry that constrain magmatic processes.^100^ China’s Tianwen-1 mission deployed the Mars Surface Composition Detector (MarSCoDe), which includes a LIBS system for in situ elemental analysis. A novel adaptive spectral drift correction method was developed to address calibration challenges arising from the Martian environment, improving quantitative accuracy of LIBS measurements.^47^ This methodological advance addresses a persistent challenge in planetary LIBS: maintaining calibration stability under variable environmental conditions and instrument aging. The Chandrayaan-3 LE-LIBS instrument demonstrated operational LIBS on the lunar surface, employing low-energy (≤4 mJ) eye-safe laser pulses at 1535 nm with an aberration-corrected concave holographic grating spectrometer achieving ≤1 nm optical resolution.^115^ The Chandrayaan-3 LE-LIBS instrument performed the first in situ LIBS analysis of lunar regolith, conducting elemental investigations near the Shiv Shakti landing point in the lunar southern higher latitude region. The instrument’s low-energy (≤4 mJ), eye-safe laser design enabled safe operation in proximity to the rover, while the aberration-corrected spectrometer achieved ≤1 nm optical resolution for accurate elemental identification.^115^

Light rare-earth elements (LREEs) are not quantifiable with conventional LIBS due to matrix effects and spectral interferences, particularly when detecting low LREE concentrations in complex geological samples. Jin et al. presented a new spectral calibration strategy for double-pulse LIBS that incorporates plasma imaging information. Figure 5a,b shows the experimental setup for in schematic form for this innovative approach. The plasma images were acquired simultaneously with the spectral signal, as shown in Figure 5c–h where the plasma is divided into 7 different brightness segments. Figure 5d shows the outermost area of plasma corresponding to the experimental environment. The double-pulse approach enhanced the emission intensity of trace LREEs in natural rock samples, improving the quantitative analysis reliability.^116^

**Figure 5:**
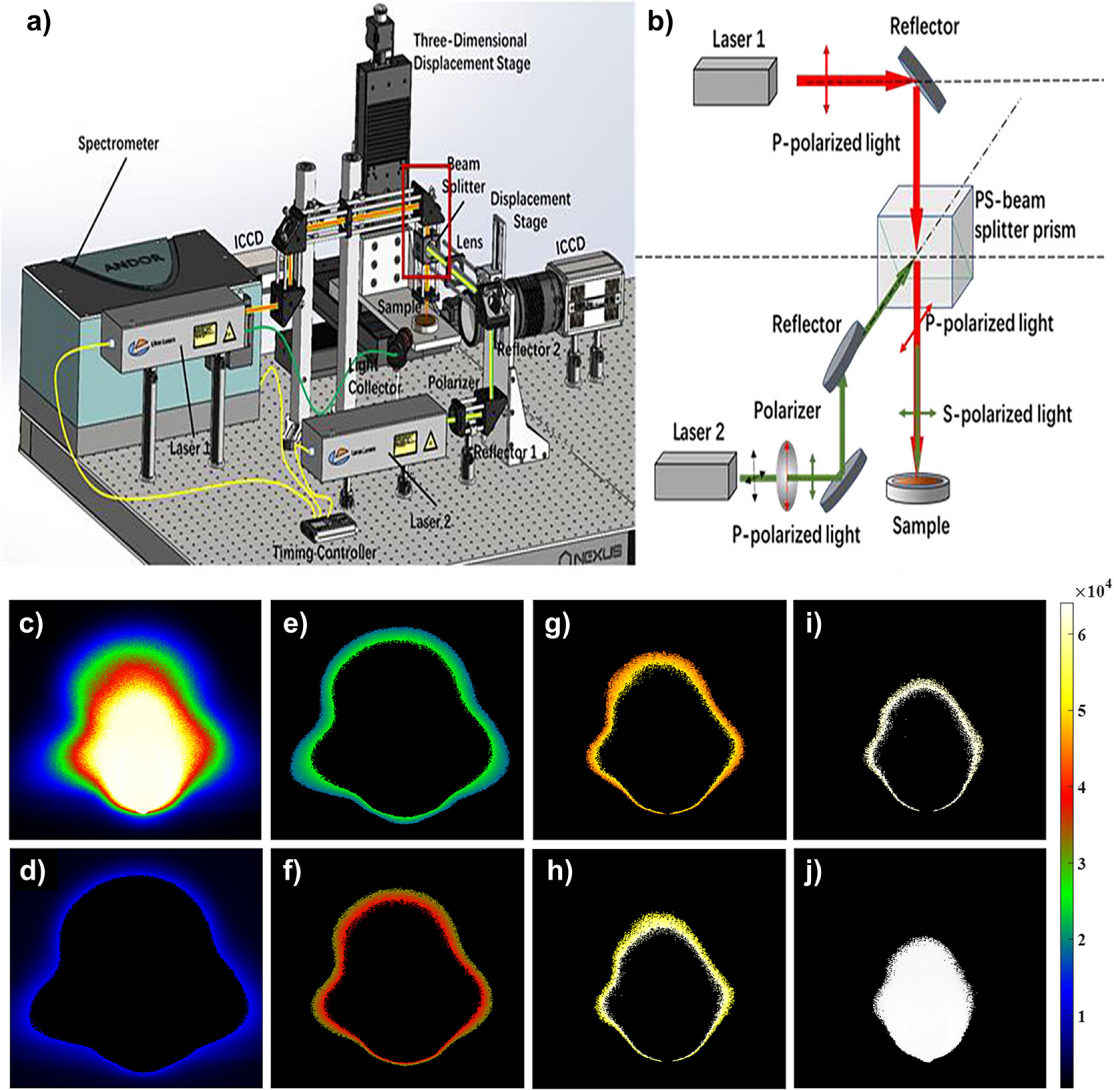
a) Schematic diagram of the DP-LIBS device experimental setup of Jin et al.^116^ b) Polarization beam-splitting scheme of the optical path of CDP-LIBS with a schematic diagram of the area division for plasma image brightness calculation. c) Plasma image acquired over the full time scale. d–j) Plasma brightness divided into seven ranges. (Adapted with permission from ref 116. Copyright 2025 American Chemical Society.)

The L3VIN (Lunar-Laser-Lab for Volatiles INvestigation) instrument’s 2D raster LIBS capability enabled elemental mapping of lunar regolith at unprecedented spatial resolution.^117,118^ Using active laser beam steering, L3VIN can acquire 20 × 20 cm elemental composition maps at 1 m distance with 1 mm/pixel resolution and 1% wt/wt detection limits.^118^ The instrument demonstrated detection of Ti in lunar analog materials including serpentine, diorite gneiss, granite, basalt, and JSC-1 regolith, with capability to detect Si at concentrations as low as 4.5 wt%.^117^ Rapin et al. reported the next-generation LIBS technique called μLIBS, which can achieve an unprecedented 50–100 μm spatial resolution with a 2-axis actuated scanning mirror for micromapping areas less than 1 cm^2^ (submillimeter-scale).^119^ Operating at a 20–50 cm standoff distance, it can map a 30 × 30 grid in under an hour, detecting minor phases down to 0.1%.^119^ This capability enables analysis of features including individual crystals, alteration phases, fracture fills, and cements in icy moons.

In the context of remote sensing endeavors utilizing rovers, it is imperative to develop portable and easily accessible spectrometers, with the capability to relay collected data back to a remote analysis station. For this, Lehner et al. introduced an autonomous in-contact sampling technique utilizing an attachable LIBS instrument.^120^ In this approach, the spectrometer module is retrieved by a Lightweight Rover Unit (LRU) at the landing site and conveyed to the designated sampling area, where a manipulative arm establishes firm contact between the device and the sample material. This in-contact method guarantees an appropriate focal distance for the spectrometer, eliminating the need for a focusing mechanism that would otherwise increase the instrument’s size and weight, while also facilitating adaptable deployment of the device without external oversight.^120^ The LRU and LIBS instrument underwent successful testing at a lunar analogue site located on Mt. Etna in Sicily.

A combination of Raman and LIBS systems has demonstrated synergistic advantages for mineral discrimination. The integration enables LIBS to provide rapid elemental screening while Raman confirms molecular identity, particularly valuable for distinguishing minerals with similar elemental compositions but different crystal structures.^94,121^ A new multimodal spectral knowledge distillation (MSKD) framework that integrates LIBS and Raman imaging has been developed, enhancing the accuracy of mineral classification via LIBS from 68% to 78% by utilizing Raman spectroscopy as the guiding method.^122^ The PHOENIX instrument, developed for lunar astronaut exploration in the Artemis program, used Raman-LIBS for rapid soil analysis and showed the instrument’s capability to identify diverse mineral phases in lunar regolith simulants.^123^ The combined approach enables astronauts to perform real-time mineralogical and geochemical analysis during extravehicular activities. Surampudi et al. also reported a hand-held Raman–LIBS instrument operating at 266 nm laser with an integrated autofocus mechanism.^37^ Figure 6a shows the instrumentation of the spectrometer and Figure 6b–d emphasizes the miniaturization of this technique enabling comfortable hand-held operation in both laboratory and field environments. The instrument functions well for distinguishing components in 1) a complex mineral–planetary simulant mixture, 2) an isotope mixture, and 3) an organic–inorganic mixture. The use of deep-UV allowed mixture detection to as low as 0.1% with no interference between the two techniques.^37^ These investigations reveal the increased robustness, interpretability, and feature discovery in mineral identification when a multimodal approach is utilized.

**Figure 6:**
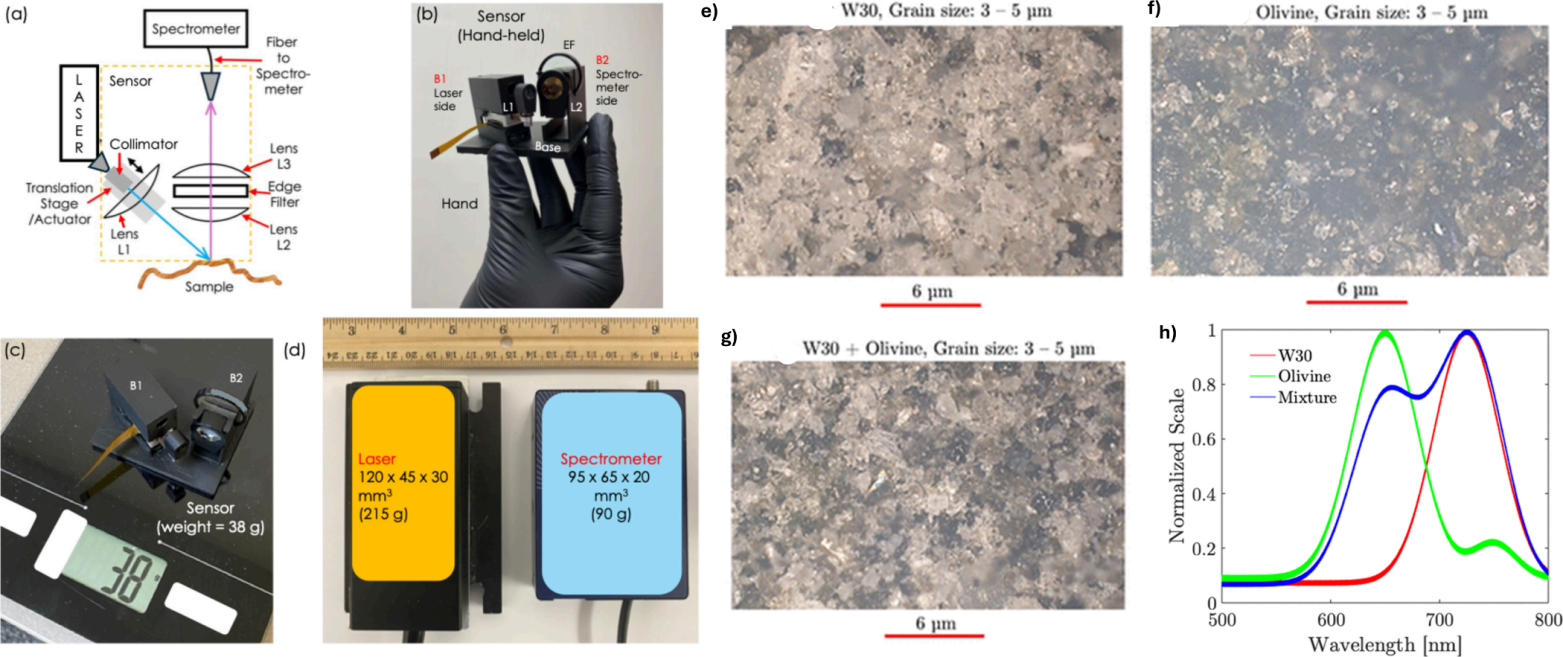
a) Schematic diagram of the PHOENIX Raman–LIBS setup. b) The hand-held sensor. c) Weight measurement of the sensor = 38 g. d) Size comparison of the entire setup with the laser and the spectrometer. Microscope images: e) sample 1: W30, f) sample 2: olivine, g) sample 3: mixture of olivine and W30. h) Fluorescence measurements with the sensor. (Adapted from ref 37. Copyright 2025 The Authors. Published by American Chemical Society under a CC BY 4.0 license.)

### 2.3 PL for Rare Earth Materials and Defects

Photoluminescence spectroscopy detects electronic transitions in minerals, providing complementary information to Raman and LIBS techniques.^124^ The method is particularly sensitive to rare-earth elements (REEs), transition-metal ions, and crystal defects that produce characteristic luminescence signatures. PL spectroscopy also detects radiation-induced defects and alteration products in planetary soils, providing insights into space weathering processes and surface exposure ages.^124^

Deep-UV photoluminescence is particularly sensitive to radiation-induced defects in silicate minerals. The 266 nm laser-based system demonstrated by Aryal et al. detected fluorescence from radiation-damaged minerals, with spectral characteristics revealing the nature of the defect centers.^51^ This capability is relevant for assessing surface exposure ages and understanding space weathering processes on airless bodies. Tucker et al. investigated various lunar regolith simulants employing distinct laser excitation wavelengths and spot sizes, revealing that configurations utilizing a 785 nm laser yielded narrow, intense fluorescence signals between 870 and 890 nm, attributed to previously unreported Neodymium as a rare earth element impurity.^125^ Lunar regolith exhibits characteristic luminescence from radiation damage accumulated over billions of years of exposure to solar wind, cosmic rays, and micrometeorite impacts. The PHOENIX instrument’s photoluminescence capability enables detection of these radiation signatures, providing insights into regolith maturity and mixing processes.^123^ Variations in luminescence intensity and spectral characteristics can reveal regions of recent regolith turnover or ancient, heavily weathered surfaces.

Notably, fluorescence from minerals is observable, particularly in the near-infrared (NIR) spectrum, where these peaks prominently emerge within an otherwise low-background emission wavelength range and are further amplified at lower temperatures.^126^ This is exemplified by the identification of NIR fluorescence from specific minerals that play a critical role in the extraction of metals, oxygen, and water from the lunar surface.^126^ Figure 6h depicts the fluorescence spectra of a pure W30 sample (Figure 6e) peaking at 655 nm, a pure olivine sample (Figure 6f) at 715 nm, and their mixture (Figure 6g) with both the peaks from W30 and olivine.

Time-resolved photoluminescence (TRPL) measurements can also discriminate between different types of radiation damage based on decay lifetimes. SuperCam’s TRPL capability represents the first deployment of this technique on Mars.^72,127^ The instrument employs time-gated detection with exposures down to 100 ns, enabling discrimination of luminescence from Raman scattering and fluorescence based on temporal characteristics.^72^ TRPL has proven valuable for detecting REE-bearing minerals, particularly phosphates such as apatite and zircon, which exhibit characteristic luminescence from Eu^3+^, Sm^3+^, and Dy^3+^ ions.^72^ The time-gated detection approach enables separation of short-lived fluorescence from longer-lived phosphorescence, enhancing specificity for different defect types.

This section shows that Raman spectroscopy, LIBS, and PL spectroscopy all provide complementary information on the inorganic contents of the soil/regolith of extraterrestrial bodies.

## 3 Tracing Water and Ice beyond Earth

The presence, distribution, and physical state of water (vapor, liquid, or ice) serve as crucial indicators of present as well as past hydrological activity, climatic conditions, and surface–atmosphere interactions. Identifying hydrated minerals or subsurface ice deposits can reveal evidence of ancient aqueous processes and guide the selection of landing sites for exploration missions.^128,129^ Works like the Mars Subsurface Water Ice Mapping (SWIM) project emphasize mapping the water–ice resources in the northern midlatitudes of Mars so that accessible ice deposits could be identified for mission landings.^130^

In recent years, NASA’s Planetary Science Division has allocated resources to various technology development initiatives aimed at facilitating forthcoming explorations of oceanic celestial bodies, including Instrument Concepts for Europa Exploration (ICEE), Concepts for Ocean Worlds Life Detection Technology (COLDTech), Scientific Exploration Subsurface Access Mechanism for Europa (SESAME), Applied Information Systems Research: Autonomous Robotics Research for Ocean Worlds (AISR:ARROW), and Astrodynamics in Support of Icy Worlds Missions.^131^

### 3.1 Raman Bands for Water–Ice and Hydrates

Raman spectroscopy provides definitive identification of water and ice through characteristic vibrational bands. Liquid water exhibits a broad O–H stretching band centered near 3400 cm^–1^, while ice polymorphs display sharper, temperature-dependent features.^132^ Beyond the ν­(OH) stretching band (∼3000–3700 cm^–1^), Raman spectra of water and ice include bending (∼1640 cm^–1^), librational (∼600 cm^–1^), and translational (∼200–400 cm^–1^) modes, with the low-frequency region being critical for identifying ice phases.^133^ These fingerprints show difference in the profile for water and ice which helps in their distinction. Liquid water exhibits broad, featureless low-frequency region while crystalline ice exhibits discrete translational and librational peaks. However, the hydrogen bonding present in these targets is a dominant control of the Raman band position which causes a redshift indicating a strong H-bond and a blueshift for a weak H-bond.^134^ One can also distinguish −OH (minerals) from water/ice by observing a sharper −OH stretch, narrow bandwidth, absence of low-frequency modes and low temperature sensitivity in the hydroxyls.^135^ Planetary bodies (Mars, Europa, Enceladus, Titan) host multiple ice types—H_2_O ice, CO_2_ ice, CH_4_ ice, and gas hydrates—that may look similar visually but differ chemically and structurally.^66,136,137^ Water ice alone has more than 17 crystalline phases with different hydrogen-bond arrangements.^138^ H_2_O ice exhibits strong O–H stretching bands (∼3090–3460 cm^–1^), CO ice depicts characteristic C=O stretching modes and CH_4_ ice shows C–H stretching modes (∼2900–3000 cm^–1^) which all fall in different spectral regions of Raman shift and makes the distinction easy.^30^

These differences produce non-overlapping spectral fingerprints, allowing clear identification even remotely. An ultracompact laser-enabled Raman spectrometer called :instrument in Situ Spectroscopic Europa Explorer” (iSEE) was developed with the objective to study ice samples in Europa.^139^ The study demonstrates the possibility of using iSEE for lunar exploration to study ice distribution from a distance to indicate the location to mission robots for drilling.^139^ The development of this instrument requires a robust fiber-coupled 515 nm laser which has been designed by the NASA Goddard Space Flight Center (GSFC) for a planetary mission with limited resources.^139^

The Raman analyzer in the Tianwen-3 mission is designed to identify hydrated minerals including gypsum, clay minerals, and other hydrous phases that record the history of water activity on Mars.^140^ The mission targets materials that preserve evidence of ancient liquid water environments, including fluvial valleys, deltas, alluvial fans, and sedimentary layers.^140^ The ExoMars Raman Laser Spectrometer (RLS) has been specifically designed to detect hydrated minerals and water ice in Martian subsurface samples.^27,141^ Veneranda et al. demonstrated RLS’s capability for recognizing wet target craters on Mars through detection of hydrated sulfates, phyllosilicates, and other water-bearing phases.^141^ The instrument’s 532 nm excitation wavelength and high spectral resolution enable discrimination of gypsum (CaSO_4_·2H_2_O) from bassanite (CaSO_4_·0.5H_2_O) and anhydrite (CaSO_4_), providing insights into past aqueous conditions.^141^ For the Martian Moons eXploration (MMX) mission, the RAX Raman spectrometer has been developed for in situ mineralogical analysis on Phobos, targeting identification of hydrated minerals and organic compounds that may reveal the moon’s origin.^36^

Shi et al. synthesized a series of magnesium chloride hydrates, MgCl_2_·*n*H_2_O (*n* = 1, 2, 4, 6, 8, 12), with pictures shown in Figure 7b, under different laboratory conditions to better understand the chloride salts identified on the surfaces of Mars and Europa.^132^ Raman spectra shown in Figure 7d, were collected over the range of 100–4000 cm^–1^ using a 532 nm laser excitation. The spectrometer was regularly calibrated to ensure high spectral accuracy. To prevent rehydration, the unstable lower hydrates (*n* = 1, 2, 4) were sealed immediately after heating, and their spectra were recorded through the sample bottle walls.^132^ In a remarkable work by Ranieri and colleagues, a new hydrogen hydrate phase was synthesized under high pressures which had twice as many H_2_ as H_2_O molecules in the unit cell and a cubic ice for the water framework.^144^ It was the first report to show the highest gas-to-water molar ratio in any crystalline solid consisting water and gas. Due to its durability at higher temperature and pressures, this gas-hydrate is expected to be present on icy extraterrestrial bodies. Raman spectroscopy was successfully applied to find spectral signatures for this phase.^144^

**Figure 7:**
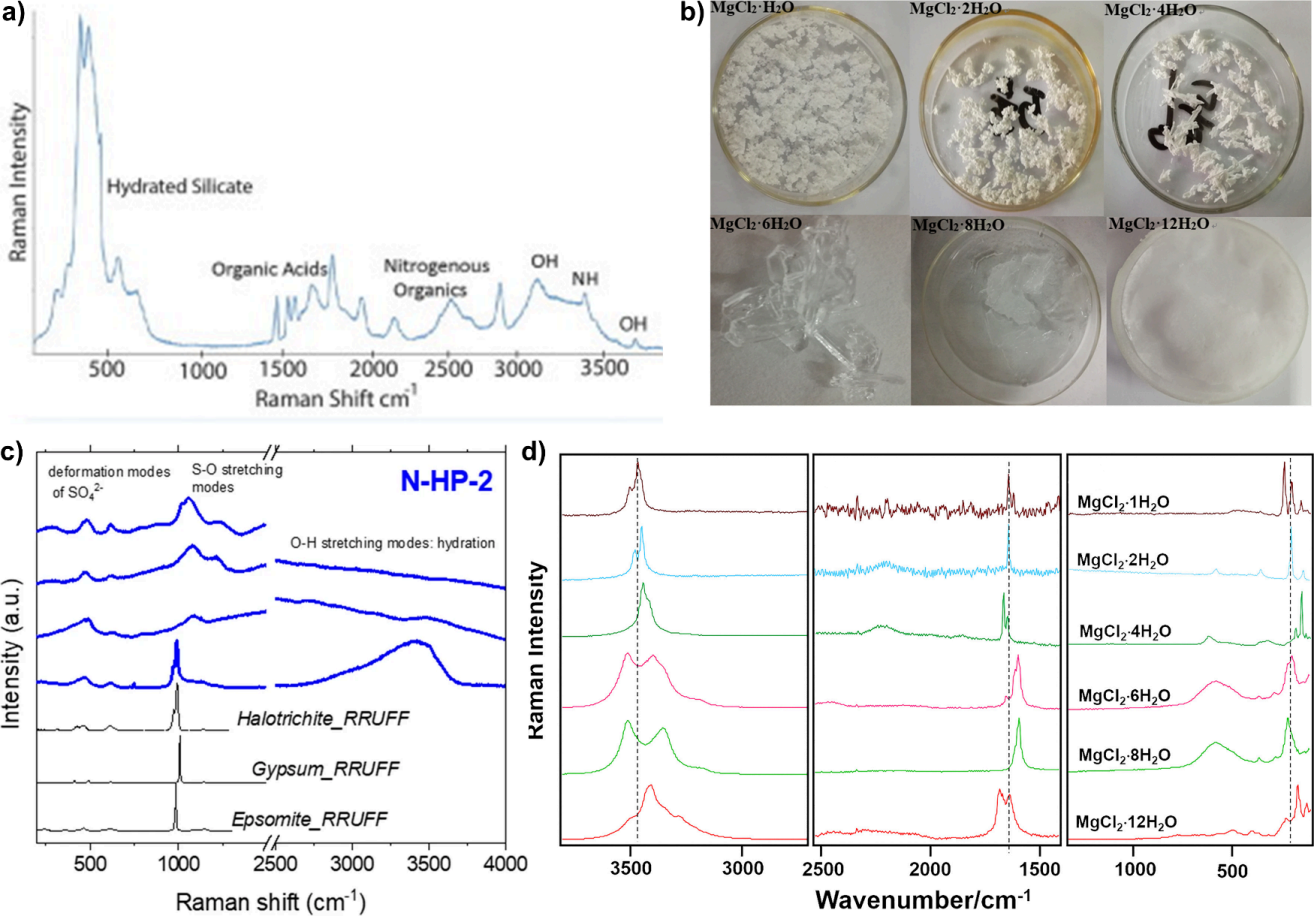
a) VIS Raman spectra of hydrated silicate, organic acids, and metal–organo compounds in volcanic sediment. (Reprinted with permission from ref 142. Copyright 2021 Elsevier.) b) Photographs of the synthesized magnesium chlorides with different hydration states (MgCl_2_·*n*H_2_O, *n* = 1, 2, 4, 6, 8, 12). (Adapted with permission from ref 132. Copyright 1999–2026 John Wiley & Sons, Inc.) c) Raman spectra of the sample of the hot spring precipitate composed of different types of sulfate-based minerals. (Adapted from ref 143. Copyright 2022 The Author(s) under a CC BY 4.0 license.) d) Raman spectra of magnesium chlorides prepared by Shi et al. with different hydration states. (Adapted with permission from ref 132. Copyright 1999–2026 John Wiley & Sons, Inc.)

In a terrestrial analog study, a Holuhraun lava sample study from the volcano–glacial region in Iceland showed the presence of hydrated silicates (possibly white micas) through Raman spectroscopy as shown in Figure 7a.^142^ In another analog study in Iceland natural specimens were gathered from three hydrothermal regions, serving as Martian analogues.^143^ The acquired Raman spectra, recorded with optical parameters akin to those of the ExoMars 2022 Raman spectrometer, demonstrated structural alterations in all secondary minerals, evidenced by peak displacements (notably in sulfur and clay minerals), modifications in relative intensity ratios (in anatase), and/or broadening of shapes (in sulfates and hematite).^143^ Figure 7c depicts the S–O and −OH stretching modes in the three different minerals in this study. The acknowledgment of silica is particularly remarkable, as it may imply the historical existence of hydrothermal hot springs characterized by nutrient abundance and redox gradients.^143^

Future missions to icy satellites will employ Raman spectroscopy as a primary tool for the ice characterization.

### 3.2 LIBS for Hydrogen

LIBS provides a direct detection of hydrogen and oxygen through atomic emission lines, enabling quantitative analysis of water content and isotopic composition. The hydrogen Hα line at 656.3 nm is particularly prominent in LIBS spectra, while oxygen lines in the near-infrared provide complementary information. The molecular structure of hydrogen can be differentiated by combining LIBS with a preheating step using a continuous-wave laser.^145^ In this approach, the sample is first heated under controlled fluence and exposure time before LIBS measurements. This technique also enables discrimination between ice and hydroxyl: in ice-containing samples, the Hα emission lines diminish after heating due to sublimation, whereas in hydroxyl-bearing materials, these signals remain largely unchanged.^145^ Figure 8 shows this phenomenon.

**Figure 8:**
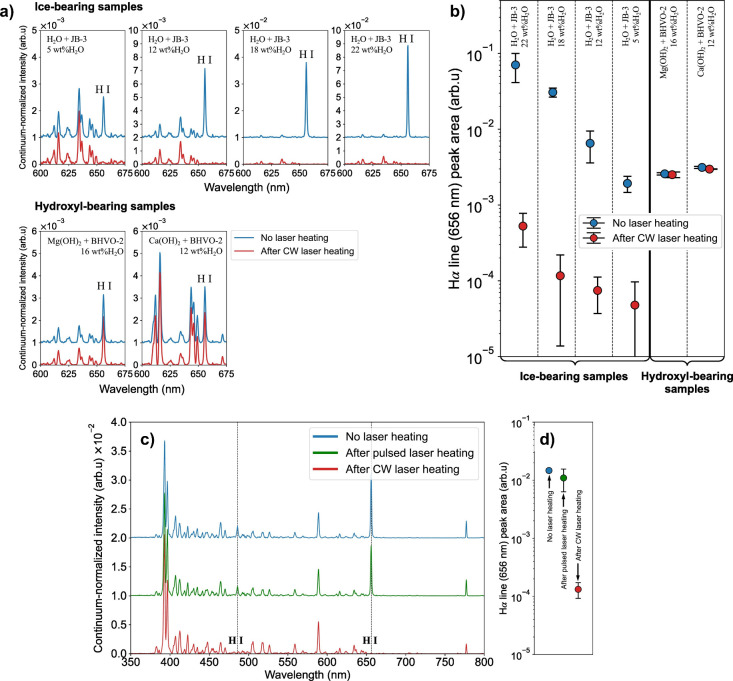
Changes in LIBS hydrogen emission after heating by a CW laser. a) Continuum-normalized spectra of ice-bearing and hydroxyl-bearing samples measured without and after heating by a CW laser. b) The Hα line peak area for each spectrum shown in panel a. c) Changes in LIBS hydrogen emission after heating by a pulsed laser. Continuum-normalized spectra of an ice-bearing sample (13 wt% H_2_O) without and after heating by a CW or pulsed laser. d) The Hα line peak area for each spectrum shown in panel c. (Reprinted with permission from^145^. Copyright 2023 Elsevier.)

SuperCam’s LIBS ability to perform depth profiling through sequential laser pulses enabled characterization of surface coatings and weathering rinds, revealing vertical variations in hydrogen content.^72^ This capability is particularly valuable for assessing recent vs ancient aqueous alteration. For lunar exploration, ISRO’s Chandrayaan-3 LE-LIBS instrument successfully operated on the lunar surface, performing in situ elemental investigations including hydrogen detection near the Shiv Shakti landing point.^115^ Another lunar exploration—the VOILA instrument on the LUVMI-X rover—was specifically configured to detect hydrogen at the lunar south pole, with spectrometer coverage optimized for the 656.3 nm hydrogen line.^146^ Laboratory measurements demonstrated the instrument’s capability to detect hydrogen in lunar regolith simulants, with quantification enabling inference of water content.^146^ The Lunar-Laser-Lab for Volatiles Investigation (L3VIN) of NASA targeted volatile compounds detection in permanently shadowed regions at the lunar south pole.^117,118^

In a terrestrial analogue study simulating Mars exploration, a LIBS–Raman sensor successfully characterized hydrated minerals in samples from the Dhofar region of Oman.^28^ The UV Raman component identified hydrated sulfates and phyllosilicates, while LIBS provided elemental context including hydrogen detection.^28^ In a similar study on Holuhraun lava samples from Iceland, strong LIBS emission features from 1470 and 1515 cm^–1^ were assigned to water molecules bound in the mineral matrix.^142^

While numerous studies have indicated favorable outcomes for detecting water–ice via LIBS under vacuum conditions, the ice-regolith regions on all terrestrial bodies are not under a vacuum. Diotte and colleagues evaluated the response of the Hα emission line in water–ice within two distinct types of ice–regolith composites through the utilization of a scanning LIBS microanalyzer.^147^ They determined that the larger particles found in wet or ice-cemented regolith, in conjunction with the laser, were the predominant factor influencing the hydrogen emission. Emission from “cemented” ice–regolith composites demonstrated an increase in Hα intensity up to approximately 15 wt%, succeeded by a decrease attributed to water saturation. Conversely, emission from “discrete” ice–regolith composites exhibited no consistent Hα response within the 0–10 wt% range.^147^ These findings underscore the necessity for calibration utilizing geological materials that accurately represent grain size, porosity, and the type of ice–regolith composite.

### 3.3 PL for Luminescence from Ice Impurities

Water and ice do not exhibit intrinsic fluorescence and, therefore, cannot be directly detected using fluorescence spectroscopy. Nonetheless, fluorescence methods can indirectly indicate the presence of water or water–ice by detecting fluorescent tracers or organic compounds embedded within icy matrices.^58^ It can reveal impurities and structural defects in ice as trace organic compounds, dissolved ions, and radiation-induced defects produce luminescence signatures that provide insights into ice formation conditions, radiation processing, and potential biosignatures. In turn, TRPL can even enhance specificity for detecting organic compounds in ice matrices.^53^

Radiolysis of water–ice generates hydrogen peroxide, ozone, and other reactive species that exhibit distinct fluorescence. This capability is particularly relevant for icy satellites exposed to intense radiation environments, such as Europa in Jupiter’s magnetosphere.^136^ Deep-UV photoluminescence offers enhanced sensitivity to aromatic organic compounds that may be present as impurities in planetary ice.^51^ The 266 nm laser-based system developed by Aryal et al. demonstrated fluorescence detection in ice samples, with capability to identify organic species including alcohols, alkanes, and amino acids.^51^ The deep-UV excitation wavelength efficiently excites aromatic compounds while minimizing interference from Raman scattering due to ice.^51^

Time-resolved luminescence measurements can also detect dissolved salts and other ionic impurities in ice. Certain ions, particularly rare earth elements and transition metals, produce characteristic luminescence that reveals ice chemistry and formation conditions. SuperCam’s time-resolved luminescence capability, while primarily applied to rock analysis on Mars, demonstrates the technical feasibility of this approach for future ice-focused missions.^72,148^

The above methods are indirect indicators of the presence of hydration on the surface of planetary bodies, so the discussion on the detection of organics/biosignatures embedded in ice is in the next section.

## 4 Detecting Chemical Signatures of Life

The presence of biomolecules like amino acids, oligopeptides, nucleobases, and fatty acids indicates a probability to find protocells, whose coexistence sets the stage for the emergence of life on an early-earth body (a terrestrial body in the early stages of geological evolution compared to the Earth).^149^ However, their coexistence on Earth or any other celestial body does not necessarily guarantee the existence of life, as the atmospheric composition and variations in the hydrochemistry on the terrestrial body decides the ultimate destiny of these prebiotic organic molecules.^149^ Their presence can be compared with the evolutionary biology of Earth and provide insights into the biology of the universe.^150,151^

The ExoMars rover and the Mars sample return (MSR) are highly important missions of the astrobiological study of our solar system (starting with Mars), whose conception began in 1996, when ESA started with the formulation of the guidelines necessary for such a study.^152^ The in situ identification of biosignatures presents considerable challenges from both scientific and technical perspectives; therefore, innovative instruments are being engineered for the purpose of detecting both extinct and extant forms of life on Mars and ocean worlds.^129,153,154^

### 4.1 Raman Signatures of Carbonates: Polyaromatic Hydrocarbons

Raman spectroscopy provides definitive identification of organic compounds through characteristic vibrational modes, including C–H stretching, C=C stretching, and functional group vibrations.^153^ The technique’s sensitivity to molecular structure enables discrimination of different organic compound classes and detection of biosignatures in geological samples.^142,155^

SuperCam’s Raman capability has been applied to search for organic compounds at Jezero Crater.^101^ The instrument’s 532 nm green laser excitation can induce strong fluorescence from certain organic compounds, potentially obscuring Raman signals.^101^ Operational strategies including deep-UV incidence, time-gated detection, and spectral deconvolution have been developed to mitigate fluorescence interference. SHERLOC’s 248.6 nm excitation wavelength efficiently generates Raman scattering while shifting fluorescence to longer wavelengths where it can be spectrally separated. The instrument has detected potential organic compounds in Martian samples, with ongoing analysis to distinguish between indigenous organics and potential contamination.^98^ A study by Corpolongo et al. demonstrated deep-UV Raman detection of kerogen in Neoarchean and Eocene microbialites using a SHERLOC analogue instrument as shown in Figure 9a,b.^156^ The research identified characteristic Raman bands from aromatic carbon structures (accumulated at different points in the sample) validating the approach for interpreting macromolecular carbon detections on Mars.^156^ This work established spectral libraries and interpretation frameworks for biosignature detection in ancient rocks.^156^

**Figure 9:**
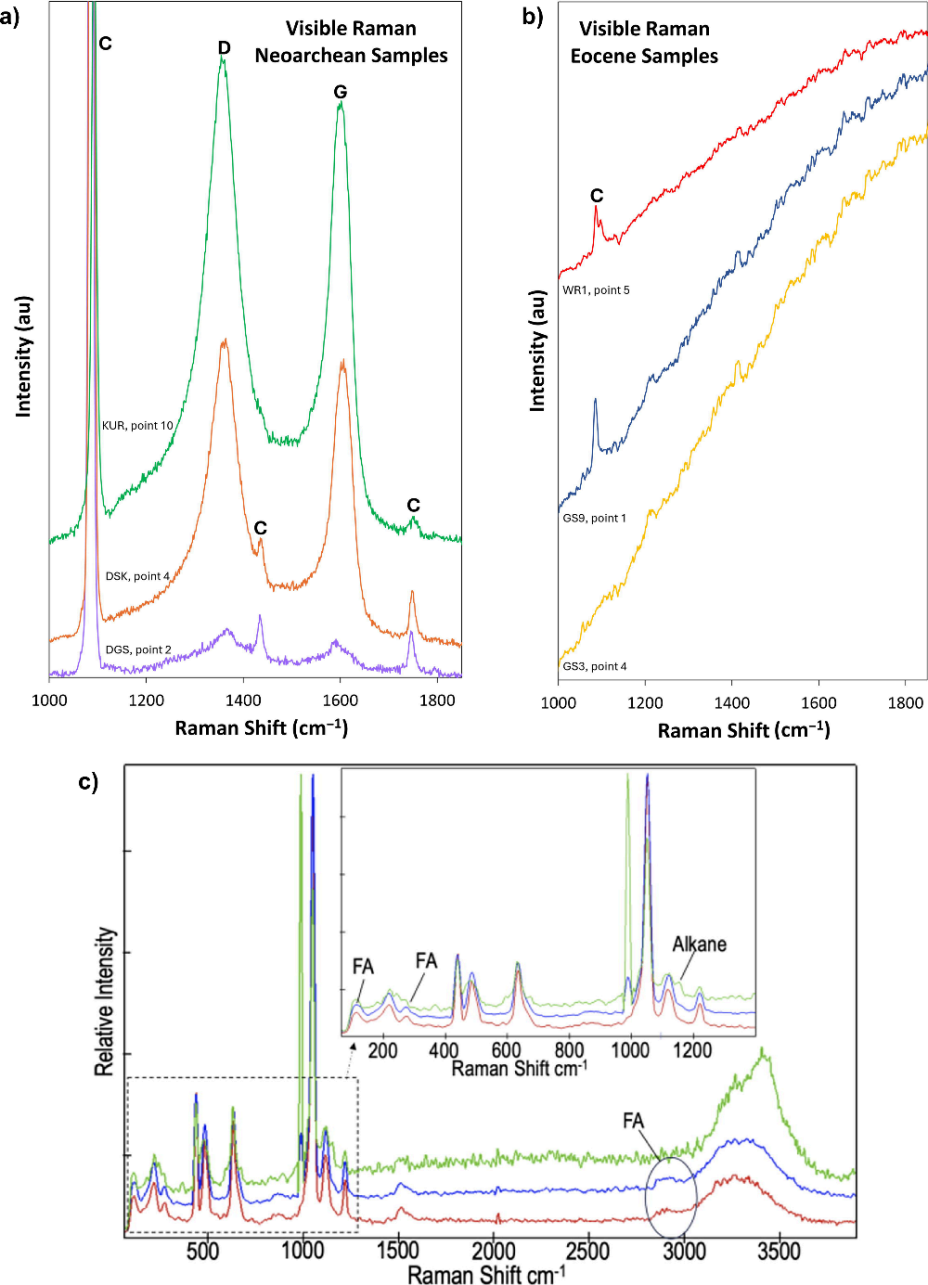
a) Three visible Raman spectra collected on the Neoarchean samples. b) Three visible Raman spectra collected on the Eocene samples. (Adapted from ref 156 under a Creative Commons CC-BY License.) c) Raman spectra showing distinct MgSO_4_·H_2_O peaks between ∼210 and 1510 cm^–1^, broad MgSO_4_·H_2_O hydration peaks between ∼3200 and 3450 cm^–1^, weaker fatty acid peaks dominated by C14:0 FA (inset), and broad/weak fatty acid peaks between ∼2850 and 2940 cm^–1^ (circled). (Adapted from ref 157 under a CC BY 4.0 license.)

Raman spectroscopy was employed by Lewis and co-workers to characterize the distribution of fatty acids and their radiolysis products on sulfate surfaces, to study organic products after γ radiolysis.^157^ Prominent Raman peaks corresponding to MgSO_4_ were prevalent in the spectra of all samples (Figure 9c), exhibiting some shifts in peak positions attributable to alterations in hydration states. The incorporation of fatty acids was rarely evident, manifesting as peaks of low intensity, and no definitive relationship between the radiation dose and the frequency of fatty acid Raman peaks was discerned.^157^ The investigation further highlighted that sample heterogeneity complicated the identification of the organic molecules. Nonetheless, Raman spectroscopic analysis successfully detected fatty acids across all radiation doses, alongside alkanes and unsaturation resulting from radiation exposure.^157^

A study examined the identification of ancient peptide folds, which are found in all life forms and thought to have emerged from the RNA–peptide world, highlighting that their identification is enhanced when attached to soil minerals, which also shield them from degradation.^158^ While the search for universal biomarkers is contentious due to the abiotic synthesis of certain biomolecules, ancient peptides are increasingly recognized as significant in the quest for extraterrestrial life, particularly on Mars, as they reveal vital insights through their interaction with minerals.^158^ The investigation of *Cryomyces antarcticus* biomolecules (biosignatures) utilizing Raman spectroscopy was conducted following exposure to varying doses of heavy ions.^159^ The Raman spectral analyses revealed a distinctive fingerprint associated with fungal colonies, characterized by two prominent peaks: a strong and broad peak within the range of 1590–1605 cm^–1^ and a secondary peak at a lower wavelength, approximately 1340 cm^–1^.^159^ The presence of an additional peak enabled the Raman analyses to differentiate melanin pigments from incinerated organic materials and/or amorphous carbon, affirming that the samples had not experienced thermal degradation and verifying the existence of C. antarcticus melanin pigments. This finding supports the hypothesis of employing this biomolecule, which is ubiquitous across all biological Kingdoms, as a biosignature in the quest for Earth-like life beyond our planet within the Solar System.^159^ Silanol has been detected by Raman spectroscopy as a structural defect of −OH groups formed by the interaction of water with crystalline quartz by Tsukada et al. indicating toward the detection of both carbonaceous organic matter and water together by Raman spectroscopy.^160^

An innovative approach, which involved sending Earth-analogue soils directly into space rather than relying solely on ground-based simulations, provided an unprecedented test of biomarker stability under authentic extraterrestrial stressors. Small fragments of Antarctic sandstones colonized by cryptoendolithic microbial communities (considered close terrestrial analogues of martian substrates) were exposed for 18 months to real space and simulated martian conditions aboard the EXPOSE-E facility in low Earth orbit.^161^ Raman analyses demonstrated that key biological signatures, including pigments such as melanin, carotenoids, and chlorophyll, as well as lipids and amino acids, remained detectable and chemically stable when shielded within mineral matrices.^161,162^ These findings underscore both the robustness of mineral-hosted biomarkers under martian-like conditions and the exceptional suitability of Raman spectroscopy for in situ life-detection strategies in future Mars exploration missions. Additionally, the NASA GSFC developed solid-state laser of 515 nm emission can be used in the iSEE instrument to study biosignatures and organics in multiple missions and locations like Europa, Mars, and ocean worlds.^139^

Terrestrial analog studies have validated Raman spectroscopy’s capability for biosignature detection in extreme environments. The ExoFiT trial in the Atacama Desert, Chile, employed representative prototypes of the ExoMars Raman Laser Spectrometer (RLS) to analyze drilled cores.^99^ Critically, the Raman systems detected organic functional groups (−C≡N, −NH_2_, C–(NO_2_)) indicative of nitrogen-fixing microorganisms, demonstrating the technique’s potential for life detection in Mars-like environments.^25,99^ Diloreto et al. performed Raman spectroscopic studies on gypsum at the hypersaline wetlands in Qatar to show the encapsulation of organic materials and subsequent preseravation by translucent gypsum.^163^ The results showed that complex Raman spectra can be linked with the indigenous microbial community, like Halobacteria and methanogenic archaea, which can be used as a standard for planetary explorations for life.^163^ Another analog study used carotenoid pigments as biogenic markers on Earth and used Raman spectroscopy to identify them in hot spring bacterial assemblages at Travertines in Italy.^164^ Microbial endolithic communities inhabiting selenitic gypsum from Eastern Poland (Badenian, Middle Miocene) were also examined using Raman microspectroscopy employing an uncommon 445 nm laser excitation for photosynthetic and photoprotective pigments.^165^ Raman analyses revealed distinct pigment signatures associated with algae and cyanobacteria, demonstrating the sensitivity of short-wavelength excitation for detecting complex organic molecules (carotenoids, scytonemin, and gloeocapsin) in mineral matrices with the first report of scytonin from natural cyanobacterial colonization in gypsum.^165^

### 4.2 LIBS Detection of Light Elements (C, H, N, O)

LIBS provides direct detection of hydrogen, carbon, nitrogen, and oxygen through atomic emission lines, enabling elemental analysis of organic compounds and assessment of C/H/N/O/P/S element ratios.^166^ The technique’s sensitivity to light elements makes it particularly valuable for astrobiology investigations.^62^

SuperCam’s LIBS system has detected carbon in Martian samples, providing insights into organic carbon content and distribution.^127^ The carbon emission line at 247.9 nm is prominent in LIBS spectra, enabling quantification of carbon abundance.^127^ Hydrogen detection through the Hα line at 656.3 nm provides complementary information, with C/H ratios potentially revealing organic vs inorganic carbon sources. Nitrogen emission lines in the UV region enable quantification of nitrogen content, with implications for understanding nitrogen cycling and potential biological processes. SuperCam has detected nitrogen in Martian samples, though interpretation is complicated by atmospheric contributions from N_2_.^127^ The L3VIN instrument’s LIBS capability targeted detection of H, C, N, and S in the lunar regolith, with implications for understanding volatile sources and potential organic compounds.^118^ Furthermore, this instrument’s 2D mapping capability enables identification of compositional anomalies that may represent organic-rich regions or volatile deposits.^118^

The process that activates and upholds potential microbial biosignatures within mineral matrices is also important in the study of astrobiology. Given that certain bacterial species are capable of facilitating the deposition of carbonates on Earth, a remote LIBS assessment of the extremophilic cyanobacterium *Chroococcidiopsis* sp. (sourced from the Nerja Cave in Malaga, Spain) was conducted under laboratory conditions that mimic Martian environments with chemical composition and gas pressure, revealing prospects for bacterial differentiation from the mineral substrate which was colonized.^167^ After a planetary analogue research at Puga Hot Spring Deposits, India, Sarkar et al. proposed a method to obtain astrobiological evidence through studying inorganics, arguing that the spectral analysis of hydrated borates coexisting with hydrous sulfates can serve as a tool for identifying fossilized or paleo hydrothermal environments on Mars that hold potential in the exploration for both extinct and extant extraterrestrial life.^168^ The advanced μLIBS’s high spatial resolution (50–100 μm) has been reported to enable detection of localized organic enrichments that may represent biosignatures.^119^ The instrument can quantify major elements (Si, Fe, Mg, Al, Ca, K, Na, Ti) and light elements (C, H, N, O, P, S) at submillimeter scales, revealing compositional heterogeneity that may indicate biological activity.^119^ Detection of minor phases down to 0.1% enabled identification of trace organic-rich phases.

Quantitative LIBS analysis of organic compounds requires careful calibration with organic standards. The development of organic-bearing calibration standards for planetary LIBS represents an active area of research, with an emphasis on materials relevant to Mars, the Moon, and icy satellites. Machine-learning approaches have been applied to LIBS spectra to improve discrimination and quantification of organic compounds.^76^

### 4.3 PL Lifetimes as Indicators of Organics

Deep-UV photoluminescence has emerged as a powerful tool for organic detection from aromatic organic compounds while minimizing interference from mineral fluorescence.^150^ PL lifetime measurements provide sensitive detection and characterization of organic compounds through their fluorescence decay kinetics. Different organic compound classes exhibit characteristic decay lifetimes, enabling the discrimination of aromatic hydrocarbons, amino acids, and other biosignature molecules.^150^ The SHERLOC device from the Mars 2020 mission has been instrumental in detecting potential organic compounds on Mars.^156^

Scheller et al. demonstrated that the emission peaks at 330–350 nm and 270–290 nm can also arise from trace amounts of Ce^3+^ associated with defects in phosphate and silicate minerals, as shown in Figure 10a.^52^ By correlating the spatial distribution of these luminescence signals with rover-based X-ray fluorescence detections of P_2_O_5_ and silicon-bearing phases, they showed that the observations can be fully accounted for by inorganic sources, although an organic contribution cannot be entirely ruled out (Figure 10b).^52^ As depicted in Figure 10c, the organics luminescence can also be accompanied by a Raman G band at ∼1600 cm^–1^ or any other organic band, thereby implying the importance of using multitechnique approach in these explorations. Sharma et al. studied organic–mineral samples from the Jezero crater of Mars in detail using Raman spectroscopy and PL spectroscopy in parallel. Figure 10d,e shows the graphitic G band and a luminescence centered at 340 nm on the SaU008 meteorite calibration target.^169^ They concluded that if photoluminescence is caused by an organic sample, it must arise from a combination of organic moieties rather than single emitters, where red/blue shifts and changes in fwhm can be observed due to the overlapping spectra of those moieties.^169^

**Figure 10:**
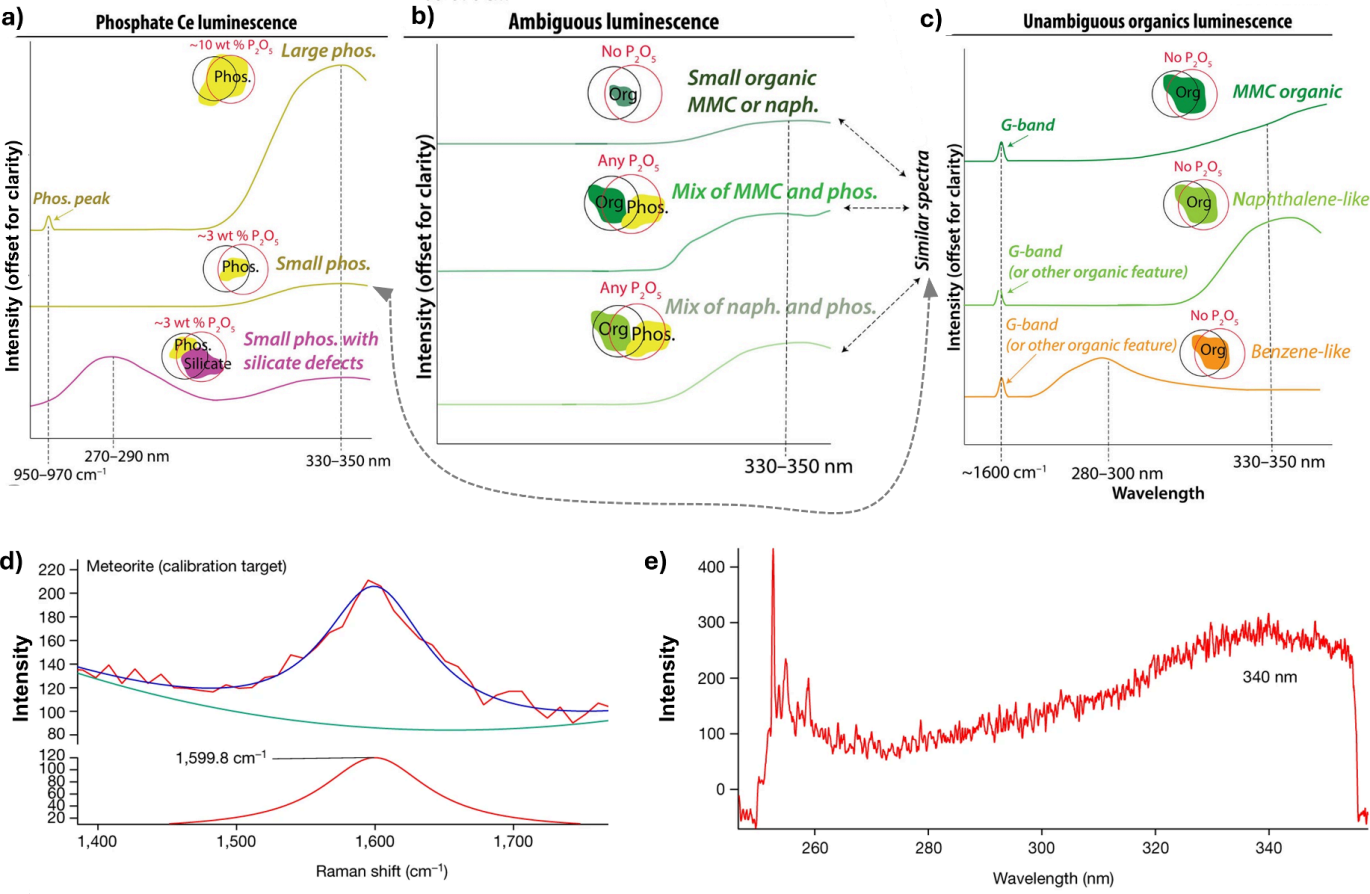
Predicted observations from a) inorganic, b) organic, and c) ambiguous sources of 330–350 nm luminescence signals. (Adapted from ref 52. Copyright 2024 The Authors, some rights reserved; exclusive licensee American Association for the Advancement of Science. No claim to original U.S. Government Works. Distributed under a Creative Commons Attribution CC BY 4.0 License.) d) Median Raman spectrum from an HDR scan on the SaU008 meteorite calibration target, which contains the known graphitic (G) band, with a Lorentzian fit. e) Corresponding average fluorescence spectrum. (Adapted from ref 169 under a CC BY 4.0 license.)

A challenge associated with the identification of biosignatures is that the biological material may be susceptible to photochemical degradation by surrounding radiation. Given that conducting sample preparation on the Martian surface is impractical, it is essential that the target material undergoes modification. Research indicated that a potential biosignature of adenosine 5′-monophosphate (AMP) adsorbed onto Ca-montmorillonite clay exhibited minimal degradation, with enhanced fluorescence indicative of the formation of 2-hydroxyadenosine, whereas exposure to UV radiation for pure AMP resulted in the cleavage of the aromatic adenine moiety.^170^ The 266 nm laser-based system developed by Aryal et al. demonstrated fluorescence detection of organic species including alcohols, alkanes, amino acids, and polymers.^51^ This deep-UV excitation wavelength efficiently excited aromatic compounds through π→π* electronic transitions, producing strong fluorescence that can be detected at trace concentrations.^51^

Time-resolved luminescence spectroscopy has also proven particularly valuable for organic detection in complex matrices. SuperCam’s TRPL capability employed time-gated detection with exposures down to 100 ns, enabling discrimination of organic fluorescence from mineral luminescence and Raman scattering.^72^ Organic compounds typically exhibit nanosecond-scale fluorescence decay, while certain minerals show longer-lived phosphorescence, providing temporal discrimination.^72^ A study by Corpolongo et al. demonstrated SHERLOC’s ability to detect kerogen fluorescence in microbialites, with spectral characteristics revealing the degree of thermal maturation and aromatic content.^156^ UV time-resolved laser-induced fluorescence spectroscopy experiments conducted on the meteorites Murchison and Allende showed the detection ability for amino acids by comparing their decay rates (1.55–3.56 ns) to those of minerals (15–70 ns).^54^ A linear relationship between the fluorescence lifetimes and the abundance of elemental nitrogen and carbon was established. These results are promising for asteroids return missions like NASA’s OSIRIS-REx and JAXA’s Hayabusa2.^54^ For future missions to icy satellites, time-resolved photoluminescence represents a key technique for biosignature detection.

Recently, a fluorescence-imager coupled with Raman spectrometer called OrganiCam was designed for detecting organic and biosignature detection on planetary bodies.^53^ This instrument used a diffused laser beam to cover a large area from a distance in meters and recorded fluorescence on half of its intensified detector. The diffuser can be removed to record Raman and fluorescence spectra from a small spot from 2 m standoff distance.^53^ Some current planetary exploration studies even focus on remote sensing of regolith through optical spectroscopic techniques with a working distance in meters to eliminate the need of sample carriage and movement on the terrain.^171^ The Ice Subsurface Habitability Investigation Penetrator (IceSHIP) platform employed a single-axis laser-induced fluorescence system for detection of low-concentration organic species, particularly amino acids—key biosignature molecules that could indicate past or present life.^172^ Unlike conventional soft-landing missions that require substantial infrastructure to access subsurface materials, penetrator missions can directly sample subsurface environments where organic compounds are better protected from radiation damage and oxidation. This capability is particularly valuable for icy worlds (Europa, Enceladus, Titan) and Mars’s polar regions, where subsurface ice may harbor preserved organic materials or even extant microbial life.

A combined Raman–LIBS–PL system in terrestrial analogue studies employed time-resolved fluorescence to discriminate organic compounds.^28^ The integration enables LIBS to detect C/H/N/O/P/S elements, Raman to identify molecular structures, and fluorescence to provide sensitive detection of aromatic compounds, offering comprehensive organic characterization.^28^ Another Raman–LIBS–PL system with high spatial and depth resolution system employing a 266 nm laser integrated in a compact package (6 cm × 3 cm × 5 cm optical head) achieved 15 μm spatial resolution and submicron depth resolution, enabling detailed analysis of lunar and planetary simulants.^51^

PL spectroscopy is particularly useful for identifying fluorescent fractions of dissolved organic matter, where cellular structures and molecules may be tagged with fluorescent dyes.^153^ However, poor staining, nonspecific binding, and mineral autofluorescence—especially from rare-earth elements—can cause false positives and negatives in biosignature detection.^172^ Unlike traditional planetary missions that depend on large soft landers for subsurface exploration, penetrator missions with miniaturized chemical instruments provide a low-cost, distributed alternative. Overall, the search for life on other celestial bodies is closely linked to the detection of water, ice, organic compounds, and minerals, as these indicators are often interconnected.^154,173^

## 5 Investigating Atmospheric Processes on Other Worlds

The knowledge of the atmospheric composition of a planetary body is highly essential for a probable manned mission and human habitability in addition to the landing and safety aspects of the missions’ rovers. Identification of gases like CO_2_, N_2_, and H_2_O could be used for in situ resource utilization (ISRU); detecting methane and other biomarker gases could indicate potential for past or present life; and understanding the seasonal variations and atmospheric dynamics could be used for planning future missions. The study of the surface of the exoplanet with respect to changing temperature and environmental conditions can provide the idea of the atmosphere of that body.^69^

The planetary spectroscopy laboratory (PSL) reported a Venus spectroscopy setup which can provide FTIR and reflectance data at temperatures up to 1000 K in the spectral range from 0.8 to 1.2 μm.^174^ These data then can be calibrated and used for future Venus missions.^174^ Similarly, new calibrated data sets can be formed for other spectroscopic studies that could indicate the real temperatures of the environment where they are operated.

### 5.1 Raman Scattering for Gas Composition

Raman scattering from atmospheric gases provides direct molecular identification and quantification without sample collection. The technique’s sensitivity to molecular vibrations enables detection of major atmospheric constituents (CO_2_, N_2_, O_2_, H_2_O) and trace species (He_2_, CH_4_, CO, H_2_S) through characteristic spectral signatures.^176^

SuperCam has demonstrated atmospheric Raman measurements on Mars, detecting CO_2_ and potentially other atmospheric species.^127^ The instrument’s remote sensing capability enabled atmospheric measurements during rock and soil analysis, providing environmental context for surface observations.^127^ While atmospheric Raman signals are weak compared to solid samples, time-averaged measurements can achieve sufficient signal-to-noise for major constituent detection.

Terrestrial analogue studies have validated Raman spectroscopy for atmospheric measurements in Mars-like conditions.^32,141^ Laboratory experiments that simulate conditions on Venus and Mars have demonstrated detection limits and spectral characteristics for CO_2_, N_2_, and trace gases.^177^ These studies inform instrument design and data interpretation strategies for planetary missions.

The development of dedicated atmospheric Raman systems for planetary missions represents an active area of research.^178,179^ Raman spectroscopy offers advantages over mass spectrometry for certain applications, including real-time measurements, no sample consumption, and sensitivity to isotopic composition through subtle spectral shifts. Future Mars missions may employ enhanced Raman systems optimized for atmospheric monitoring, enabling the detection of seasonal variations and potential biosignature gases such as methane.

### 5.2 LIBS Plasma Emission for Volatile Detection

LIBS plasma emission from atmospheric gases provides elemental analysis of atmospheric composition with sensitivity to major constituents and trace species. Laser-induced plasma ablates and excites atmospheric molecules, producing atomic emission lines characteristic of the constituent elements.^180^

SuperCam’s LIBS measurements on Mars include atmospheric contributions, particularly for major elements such as carbon and oxygen from CO_2_.^127^ The instrument’s microphone records acoustic signals from laser-induced plasma, with acoustic characteristics revealing atmospheric pressure and composition.^127^ This acoustic approach provides complementary information to spectroscopic measurements, enabling real-time atmospheric monitoring. LIBS detection of atmospheric water vapor represents a valuable capability for planetary exploration. Hydrogen and oxygen emission lines in LIBS spectra reveal atmospheric humidity, with implications for understanding diurnal and seasonal water cycles. SuperCam has detected variations in atmospheric water content at Jezero Crater, providing insights into Martian atmospheric dynamics.^127^

Lunar LIBS applications have focused on volatile detection and resource assessment. The VOILA instrument on the LUVMI-X rover was specifically designed for detecting hydrogen at the lunar south pole, with a spectrometer covering 350–790 nm to capture the hydrogen line at 656.3 nm along with major rock-forming elements.^146^ Laboratory measurements with a breadboard setup verified the instrument’s capability to detect and quantify hydrogen, enabling inference of water content in lunar regolith.^146^ The Diode Laser Spectrometer (DLS-L) is the key instrument onboard the Luna-27 lander, designed to directly probe the Moon’s geological and geochemical structure through in situ isotopic measurements of H_2_O and CO_2_ released from subpolar lunar regolith.^181^ By performing pyrolysis-based isotopic analysis of volatiles, DLS-L enables the first contamination-free determination of D/H, ^18^O/^17^O/^16^O, and ^13^C/^12^C ratios in lunar soil, providing a unique window into the origin and evolution of lunar volatiles.^180,181^

It was possible to detect aerosol particles in earth’s atmosphere by optical trapping with a combined Raman–LIBS system.^182^ Although this study is aimed at pollution detection and quantification of aerosols of marble, gypsum, baking soda, and activated carbon adsorbed potassium bicarbonate, it has applications in providing qualitative and quantitative assessment of an extraterrestrial body.^182^ The 2024 review by Sun et al. provides a comprehensive idea of the capabilities of LIBS in detecting atmospheric aerosols, particulate matter and even isotopes. Low particle concentrations and complex spectra were considered as existing hindrances to signal intensity and stability, thereby affecting sensitivity and accuracy.^64^

Future applications of LIBS for atmospheric analysis include efficient detection of trace gases and aerosols. The technique’s sensitivity to dust and aerosol composition enables characterization of atmospheric particulates, revealing sources and transport processes. Development of time-resolved and ultrafast LIBS methods may enhance sensitivity to trace atmospheric species, enabling detection of potential biosignature gases.

### 5.3 PL Quenching/Lifetime Studies for Reactive Gases

Photoluminescence quenching and lifetime measurements provide indirect but sensitive detection of atmospheric gases through their interactions with luminescent species.^183^ Gas-phase quenching of fluorescence and phosphorescence reveals atmospheric composition and pressure, while lifetime variations indicate specific molecular interactions.^184,185^

SuperCam’s TRPL capability enables measurement of luminescence decay lifetimes, which are sensitive to atmospheric pressure and composition.^72^ The SHERLOC spectrometer on the Perseverance rover showed Raman and fluorescence background spectral signatures when measurements were taken targeting the nighttime sky of Mars.^186^ These background spectral signatures could also provide information about the atmosphere, and the types of gases present there. Nonetheless, the removal of these background spectra is essential to obtain a high (signal-to-noise) S/N ratio to analyze the target material correctly. One of the Tianwen-3 mission’s nine scientific themes focuses on the “spatiotemporal evolution of water and volatiles on Mars”, aiming to determine water and volatile contents in various materials and measure their isotopic compositions (particularly hydrogen, chlorine, and sulfur isotopes) to trace evolutionary history.^140^ This approach recognizes that hydrogen isotopes preserved in hydrated minerals provide critical constraints on atmospheric loss processes and the fate of Mars’s ancient water inventory.

In addition to the direct study of the soil, NASA Langley has utilized planar laser-induced fluorescence as an experimental diagnostic tool to visualize and measure the exhaust flow field when a lander’s engine fires close to a planetary surface.^187^ In addition to plume impingement they have also reported the large-scale usage of this technique to visualize and measure complex, highly compressible flow structures such as shock interactions, mixing, and unsteady wake behavior which form behind the spacecrafts traveling at hypersonic speeds.^187^

PL has potential applications in studying atmospheric aerosols and organic hazes on planetary bodies. Organic haze particles, such as Titan tholins, contain aromatic and conjugated compounds that exhibit characteristic fluorescence under deep-UV excitation, aiding compositional analysis.^188,189^ Future missions to Titan and similar worlds may explore PL lifetime measurements for detecting organic gases since gas-induced fluorescence quenching can reflect molecular interactions and atmospheric composition.

## 6 Summary

Through our comprehensive literature review it can be stated that the optical spectroscopic techniques of Raman spectroscopy, laser-induced breakdown spectroscopy, and photoluminescence spectroscopy are versatile, posing utility in the sensing/detection of inorganic compounds, water/ice deposits, organic forms, and atmospheric conditions of an extraterrestrial body. They show to be complementary to each other in obtaining qualitative and quantitative information on the target samples and together they have the capability form a highly effective triad—Raman–LIBS–PL—for planetary exploration missions. Table 2 summarizes the results of this Review and shows the chemicals/compounds which can be detected by each technique.

**Table 2:** Capabilities of Raman, LIBS, and PL Spectroscopies in the Detection of Different Materials of Interest in Planetary Exploration Missions

**Application Area**	**Raman Spectroscopy**	**Laser-Induced Breakdown Spectroscopy (LIBS)**	**Photoluminescence (PL)**
**Inorganics/Minerals**	• Silicates (olivine, pyroxene, feldspar)	• Elemental composition (quantitative: ±5–10%)	• Transition metal ions (Mn^2+^, Fe^3+^, Cr^3+^)
	• Carbonates (calcite, dolomite, siderite)	• Major elements: Si, Al, Fe, Mg, Ca, Na, K, Ti	• Rare earth elements (Eu^2+^/^3+^, Sm^3+^, Dy^3+^)
	• Sulfates (gypsum, jarosite, alunite)	• Minor/trace elements: Mn, Cr, Ni, Zn, Cu, Sr, Ba	• Defect centers in minerals
	• Phosphates (apatite, whitlockite)	• Light elements: H, C, N, O	• Thermal history indicators
	• Oxides (hematite, magnetite, ilmenite)	• Stoichiometric ratios (Mg/Si, Ca/Si, Fe/Mg)	• Radiation exposure history
	• Amorphous phases (opal, volcanic glass)	• Mineral group identification via elemental ratios	• Alteration state indicators
	• Crystal structure information	• Geochemical indices (CIA, Mg#)	
	• Phase identification		
	• Polymorphs discrimination		

**Water/Hydration**	• Structural water in minerals (qualitative)	• Hydrogen detection (H I lines: 656.3 nm)	• Indirect hydration information
	• OH stretching modes (3000–3700 cm^–1^)	• Isotopic ratios: D/H (±10–20% precision)	• OH-related luminescence
	• H_2_O bending modes (∼1630 cm^–1^)	• Oxygen isotopes: ^18^O/^16^O (limited)	• Water-sensitive emission changes
	• Phyllosilicates (clays, micas)	• Quantitative H abundance	• Hydration state affects PL intensity
	• Hydrated sulfates and carbonates	• Elemental indicators of hydration	
	• Water ice (crystalline vs amorphous)		
	• Relative hydration state		

**Organics/Biosignatures**	• Molecular structure (qualitative)	• Elemental composition (C, H, N, O, P, S)	• Fluorescent organics detection (high sensitivity)
	• C–C, C=C, C–H bonds	• C/H, C/N, C/O ratios	• Aromatic compounds (PAHs, proteins)
	• Aromatic compounds (PAHs)	• Quantitative carbon content	• Amino acids (tryptophan, tyrosine, phenylalanine)
	• Aliphatic chains	• Bioessential elements (P, S)	• Chlorophyll and porphyrins
	• Functional groups (limited)	• Detection limits: ∼100 ppm for C	• Humic substances
	• Deep-UV Raman (248.6 nm) for organics		• Kerogen and bitumen

**Atmosphere/Environment**	• Background atmospheric signatures	• Aerosol/dust elemental composition	• Indirect gas detection via quenching
	• Dust/aerosol particles (if deposited)	• Suspended particle analysis	• O_2_ quenching of luminescence
		• Atmospheric dust chemistry	• Pressure-sensitive emissions
		• Difficult plasma formation in low pressure)	• Aerosol particle luminescence

### 6.1 An Era of Raman Spectroscopy

From the bulk of reports focusing on remote sensing through Raman spectroscopy, it can be concluded that it is the most sought-after technique in planetary exploration missions, thanks to the simplicity and versatility of the technique.^190–196^ Some of Raman spectroscopy’s major advantages are as follow:

i. High specificity combined with spatial resolution at the micrometer scale makes it well suited for heterogeneous planetary materials such as breccias, regolith, and sedimentary rocks.
ii. The ability to acquire data through transparent matrices and coatings enables in situ analysis of inclusions and fine-grained phases that are often inaccessible to bulk analytical techniques.
iii. High compatibility with miniaturization has led to its successful deployment on rover-based instruments designed for Mars exploration.
iv. Insensitivity to grain size and surface roughness, so no sample preparation is required in the field.
v. Ability to link spectral features to crystal structure and molecular bonding, unlike reflectance- or emission-based methods.

These characteristics make Raman spectroscopy exceptionally powerful for rapid, reliable geological interpretation and for supporting the search for past or present life during robotic planetary missions.^60^ It has been almost 100 years since the discovery of the phenomena of inelastic scattering of light, Raman effect, by the Indian physicist Dr. C. V. Raman. The advancements in Raman techniques are owed to the last century’s scientific works leading to a significantly higher efficacy of Raman in remote sensing applications whether on earth or on extraterrestrial projects.^33,37,94,97,186,197,198^ Table 3 summarizes some of the recent reports which justify Raman spectroscopy as a must-have technique for planetary sensing applications.

**Table 3:** Advancements in Raman Spectroscopy for Remote Sensing in Planetary Exploration

**Advancement**	**Description**	**Impact on Planetary Exploration**	**Refs**
Instrument miniaturization	Development of compact, low-mass, low-power Raman systems	Enabled rover-based Raman instruments for Mars exploration	^199^,^200^
In situ, nondestructive analysis	No need for sample preparation or alteration	Preserves geological context and supports rapid decision-making	^165^
High mineralogical specificity	Sensitivity to molecular vibrations and crystal structure	Accurate identification of minerals, polymorphs, and amorphous phases	^29^,^33^,^35^
Micrometer-scale spatial resolution	Laser spot sizes of a few micrometers	Effective analysis of heterogeneous rocks and fine-grained materials	^26^,^51^,^96^
Operation under planetary conditions	Robust performance under low pressure and extreme temperatures	Reliable measurements on Mars-like environments	^96^,^123^
Reduced matrix dependence	Spectral features linked to structure rather than bulk chemistry	More robust than elemental-only techniques	^27^
Synergy with complementary techniques	Integration with LIBS, VIS–IR, and imaging systems	Comprehensive mineralogical and geochemical interpretation	^38^,^94^,^97^

Interpreting Raman data in planetary exploration requires careful distinction between primary biosignatures and alteration products, especially for organics and biomolecules as weathering and radiation (UV, cosmic rays, charged particles) may lead to the degradation the organics (chemical alteration, hydration/dehydration, oxidation) and mimic abiotic compounds or induce phase transformation, oxidation, hydration/dehydration, and radiolysis amorphization.^201^ Although the −OH stretching from the hydrates is retained even in extreme conditions, radiation damage may lead to altercation in the hydrogen bonding fingerprints of the hydrates along with loss of spectral sharpness.^143^

Despite its strengths, Raman spectroscopy alone cannot deliver a complete physicochemical characterization of planetary materials. Its limited sensitivity to elemental abundances, susceptibility to fluorescence interference, and reduced performance for weak scatterers or complex surface coatings constrain standalone interpretation. An advanced version of Raman spectroscopy that can detect trace elements, organics, and biosignatures with a strong signal strength (10^6^–10^10^ times) has been developed in the past decade called surface-enhanced Raman spectroscopy (SERS).^202^ While laboratory studies demonstrate its power for trace detection, translating SERS into robust, flight-qualified instrumentation remains difficult under planetary conditions such as radiation, temperature extremes, and dust exposure.^203^

### 6.2 The Triad of Raman–LIBS–PL

Table 4 puts together the missing links and research gaps in the utilization of Raman, LIBS, and PL spectroscopies for sensing applications on extraterrestrial bodies.

**Table 4:** Missing Links and Limitations of Each Spectroscopic Technique for the Reported Applications

**Application Area**	**Raman Limitations**	**LIBS Limitations**	**PL Limitations**
**Inorganics/Minerals**	• Fluorescence interference (esp. organics)	• Cannot distinguish polymorphs	• No molecular structure and crystal structure information
	• Cannot determine trace elements	• Matrix effects (±5–10% accuracy)	• Limited mineral coverage
	• Limited depth penetration	• Spectral complexity (line overlap)	• Cannot determine composition alone
		• Surface Destructive (microablation)	• Emission overlaps are common

**Water/Hydration**	• Qualitative only (no quantitative H_2_O wt%)	• Isotopic precision limited (±10–20%)	• Indirect detection only
	• Limited depth profiling		• Low specificity for water
			• Cannot quantify water content

**Organics/Biosignatures**	• Fluorescence overwhelms Raman signal	• Destroys sample (ablation)	• Cannot identify specific molecules
	• No quantitative organic abundance	• Limited functional group information	• Background fluorescence interference
	• Cannot detect all organic types	• Detection limits higher than PL (100 ppm)	• False positives from minerals possible

**Atmosphere/Environment**	• Weak Raman cross-sections for gases	• Difficult plasma formation in low pressure	• Indirect detection only
	• Requires high gas concentrations	• Limited gas detection capability	• Limited gas specificity
	• Limited atmospheric applications	• Aerosols only (not gases)	• Quenching effects complex
	• MISSING: Direct atmospheric gas quantification	• MISSING: Direct atmospheric gas quantification	• MISSING: Direct atmospheric gas quantification

Table 4 serves as a critical directive for planetary mission planning. While Raman spectroscopy, LIBS, and photoluminescence spectroscopy are powerful techniques that have revolutionized planetary exploration, each technique has well-defined limitations that constrain its applicability:

- Raman: excellent for structure, poor for composition.
- LIBS: excellent for composition, poor for structure.
- PL: excellent for sensitive detection, poor for identification.
- All three: ineffective for direct atmospheric gas quantification.

From Tables 2–4, the key message is clear: multi-technique integration is a scientific necessity. Successful planetary missions must carefully design instrument suites that address the limitations of individual techniques through strategic complementarity. Table 4 provides mission planners with a systematic framework for identifying these limitations.

The choice of the laser wavelength plays an essential role in the effectiveness of the characterization technique and the instrument.^204,205^ Raman scattering intensity scales with the fourth power of excitation frequency, making deep-UV excitation ∼300× more efficient than near-infrared. Fluorescence interference, a persistent challenge in planetary materials containing organic matter, transition metal ions, and structural defects, varies dramatically with wavelength. Spatial resolution, penetration depth, and photochemical damage risk all depend critically on wavelength selection. Table 5 shows wavelength-dependent trade-offs for the five key performance metrics identified in the literature, namely Raman scattering efficiency, fluorescence suppression, penetration depth, spatial resolution, and photochemical damage risk.

**Table 5:** Wavelength-Dependent Trade-Offs of the Three Techniques—Raman Spectroscopy, LIBS, and PL Spectroscopy—for Sensing Applications

**Wavelength Region**	**Raman Scattering Efficiency (vs 1064 nm)**	**Fluorescence Suppression**	**Penetration Depth**	**Spatial Resolution**	**Photochemical Damage Risk**	**Refs**
**Deep-UV**	∼300× higher^203^	Excellent	Low (strong absorption)	Very High (sub-μm)	Moderate (bond breaking)	^34^,^37^,^206^
**UV**	∼100–200× higher^203^	Good (time-gating)	Medium	High	Moderate	^135^,^173^,^201^
**Visible**	∼20× higher^203^	Good (time-gating)	Medium	Medium–high	Low (below damage threshold)	^207^
**NIR**	∼5–10× higher^203^	Fair (reduced fluorescence)	Medium–high	Medium	Low	^83^,^132^
**IR**	Baseline (1×)^203^	Poor (but organics minimal)	High (ice penetration)	Medium (LIBS spot)	Very low (thermal only)	^24^,^48^,^81^

According to Table 5, each wavelength regime provides advanced sensing capabilities for specific techniques and target materials. These are discussed below:

i. **Deep-UV Regime**: Resonance Raman effects occur when excitation wavelength matches electronic absorption bands of aromatic compounds, polycyclic aromatic hydrocarbons (PAHs), and conjugated organics, increasing Raman intensities by factors of 10^2^ to 10^6^.^135,206^ This phenomenon is well exhibited by deep-UV excitation of 248.6 nm on the SHERLOC system on the Mars 2020 Perseverance rover, which has enabled the detection of trace organics even in the presence of strong mineral fluorescence that would have otherwise overwhelmed visible-wavelength Raman.^98^ The compact 266 nm Raman–LIBS sensor represents a recent innovation integrating both Raman and LIBS in a single instrument using a frequency-quadrupled Nd:YAG laser (1.5 ns pulse width, 10 mW average power) and has manifested higher scattering from isotope mixtures (D_2_O in H_2_O) enabled 0.1 vol% detection limits with low spectral resolution (20 cm^–1^), previously achievable only with bulky intensified CCDs at visible wavelengths along with reduced fluorescence.^37,51^

ii. **UV Regime**: 355 nm PL exploits the third harmonic of Nd:YAG lasers for time-resolved fluorescence spectroscopy, which can be used for detecting rare-earth ions exhibiting fluorescence with lifetimes of microseconds to milliseconds.^68,208^ Aromatic compounds and PAHs also exhibit strong fluorescence under 355 nm excitation, with emission spectra providing structural information complementary to Raman.^201^
iii. **Visible Regime**: A 532 nm green laser is the most widely deployed wavelength in planetary spectroscopy, employed by SuperCam (Mars 2020), RLS (ExoMars), and numerous laboratory systems.^27,148,194^ Time-gating with an intensified CCD (gate width down to 40 ns) removes ambient light and mineral fluorescence, enabling daytime operation on Mars. Major mineral groups are detectable at 5–10% abundance within 0–12 m range.^209,210^ The 532 nm wavelength balances Raman efficiency (∼20× higher than 1064 nm) against fluorescence risk, with detector response maximized in the visible range.^210^ Critical CH and OH functional groups (2800–3650 cm^–1^ Raman shift) require visible excitation to avoid detector sensitivity drop-off in the near-infrared.^103^
iv. **Near-Infrared Regime**: 785 nm Raman spectroscopy is widely used in laboratory settings to minimize fluorescence from organic-rich samples.^83^ In terms of planetary studies, tar-containing carbonates from Devon Island (Canadian High Arctic) demonstrated that 785 nm excitation minimized fluorescence, revealing dolomite fingerprints obscured at intermediate wavelengths.^151^ The discovery of compact, powerful, stable, and reliable NIR solid-state laser sources has played an important role in the instrument miniaturization of Raman systems and their portability.^39^ However, NIR wavelengths sacrifice the Raman efficiency for fluorescence suppression. This trade-off is favorable only when fluorescence is severe and cannot be mitigated by time gating or sample preparation.
v. **Infrared Regime**: 1064 nm is the standard wavelength for laser-induced breakdown spectroscopy, employed by SuperCam, ChemCam (Curiosity), MarSCoDe (Zhurong), and most laboratory LIBS systems.^121,122,192,211^ It is considered for combined LIBS-Raman systems to use a single laser for both techniques. However, the fourth power law makes 1064 nm Raman ∼20× less efficient than 532 nm and ∼300× less efficient than 250 nm.^32^ A Raman band at 4000 cm^–1^ appears at 1852.5 nm when excited at 1064 nm, where detector response is ∼60× weaker than at visible wavelengths.^32^ These factors led to abandonment of 1064 nm Raman in favor of dual-laser systems (1064 nm LIBS + 532 nm Raman).^211,56^

vi. **Long-Wave Infrared**: LWIR-LIBS is an emerging technique combining conventional UV/vis/NIR LIBS with mid-infrared spectroscopy (2500–700 cm^–1^) to detect molecular fragments, vibrational transitions, and specific bonds. Applications include PAH detection (anthracene) and carbon microstructure characterization under helium and argon atmospheres.^213,214^

To fully exploit the capabilities of each technique used in planetary exploration missions, it is essential to develop a thorough understanding of their instrumentation requirements and optimize them so that multiple techniques can efficiently share common resources. This approach has already proven successful on Mars 2020 Perseverance and will continue to guide the design of future planetary exploration missions to Mars, the moon, Europa, Enceladus, Titan, and beyond. Table 6 reports some of the recent published works on this multitechnique approach for remote sensing. The literature shows that this approach mitigates most of the challenges related to the space exploration missions described in the next section.

**Table 6:** Some Recent Reports of Multitechnique Approachess Used for Remote Sensing Application

**Multitechnique Approach**	**Target Application**	**Year**	**Ref**
VIS–NIR–LIBS–Raman	Analysis of the acid alteration of volcanic deposits	2025	^214^
Raman–XRD–Mossbauer	Detection of the presence of microbial iron reduction samples on rocks	2025	^215^
IR–LIBS–Raman	In-depth characterization of the geology and mineralogy of the Jezero crater on Mars	2022	^24^
Raman–LIBS	Analysis of material composition of minerals	2021	^121^
Raman–LIBS	Mineral classification	2025	^122^
Raman–LIBS	Measurement of geometrical topography and elemental and molecular information	2023	^96^
Raman–LIBS	Hemlholtz ARCHES Project - Geochemical testing	2024	^216^
Raman–LIBS–PL	Detection of explosives	2019	^217^
Raman–LIBS–PL	Detection and quantization of organics and inorganics in the soil	2025	^51^
Raman–LIBS–PL	Combining new mineral models for advanced exploration and visualization of critical raw materials -EU Horizon DEXPLORE project	Started in 2025	^210^

In our experience with the EU DEXPLORE project, where we integrated Raman, LIBS and TRPL techniques to obtain a platform for the stand-off identification and characterization of mineral phases relevant to Critical Raw Materials (CRM) and Strategic Raw Materials (SRM) exploration, we observed that the efficiency and successful sensing by the system depends on various design choices, like excitation setup, optical setup, collection system, and the detection system.^210^ Out of these, the excitation subsystem represented the most critical design choice, as the three implemented techniques impose partially conflicting requirements for maximum efficiencies. As discussed before, LIBS systems typically use 1064 nm (Nd:YAG) infrared excitation, where emission occurs mainly in the UV–visible range, requiring UV–visible detectors while, Raman spectroscopy produces signals close to the excitation line; using 1064 nm would shift Raman signals into the near-infrared, necessitating InGaAs detectors instead of standard silicon sensors. TRPL imposes stricter requirements, as excitation must occur above the material’s band gap to enable radiative recombination; thus, 1064 nm is generally unsuitable and would severely limit measurable materials. It would also require multiple detector types, complicating the system. An intermediate 785 nm option allows single silicon-detector use for LIBS (200–750 nm) and Raman but suffers from reduced detector sensitivity in the near-infrared and does not meet TRPL performance needs. Therefore, 532 nm excitation (Nd:YAG second harmonic) was selected as the optimal compromise. It supports efficient LIBS detection in the deep UV (200–350 nm), where key elements (e.g., Fe, Mg, Ti, Au, Ag) emit strongly, while remaining ideal for Raman with mature silicon-based detection and available optics. Shorter wavelengths were avoided to limit fluorescence interference. The system uses a pulsed 532 nm laser (∼60 mJ, ∼8 ns, 1–20 Hz).^210^

The studies in Table 6 have also utilized a combination of different remote-sensing and spectroscopic techniques to achieve a more comprehensive analysis of materials relevant to planetary science. This multi-instrument approach provides complementary information, overcoming the limitations of any single technique enabling a more robust characterization of the target materials, but challenges like fluorescence interference, cross-instrument calibration, and sample heterogeneity can persist.^175,214^ Therefore, it becomes necessary to consider various trade-offs between the complementary techniques, as discussed above, when developing combined techniques for planetary explorations.

## 7 Challenges and Mitigation

All the remote sensing technologies deployed or conceived for the application in planetary exploration objectives are constrained by several technical, environmental, and operational challenges. The principal challenges which verify the eligibility of a technique to be deemed deployable for exploration missions are as follows:

i. *Space and Mass Constraints*: Planetary missions impose strict limits on instrument mass, volume, and power consumption as each additional gram directly impacts launch costs and mission design.
ii. *Harsh Environmental Conditions*: Extreme temperatures, vacuum or low-pressure atmospheres, radiation exposure, and mechanical stresses during launch and landing can degrade instrument performance or cause failure.
iii. *High Mission Costs*: Budgetary constraints limit payload complexity and redundancy, restricting the number of instruments that can be deployed.
iv. *Geochemical Complexity*: Planetary materials are heterogeneous, and what is found on Earth will not necessarily be found on other planetary bodies.
v. *Data Complexity and Quantification Challenges*: Spectral interference, matrix effects, signal drift, and nonlinear responses complicate quantitative interpretation, especially under variable working distances and environmental conditions.
vi. *Analytical Limitations of Single Techniques*: Individual spectroscopic methods provide incomplete information—LIBS excels in elemental analysis, Raman in molecular and structural identification, and PL in electronic and defect-related properties.

These challenges must be addressed before launching a new mission to the space as the research field of planetary exploration is high risk–high gain. The mitigation techniques must not add to the existing challenges nor should create a new problem to solve. Over the years, a combination of engineering innovations and methodological strategies has been developed to mitigate these limitations, which are discussed below:

i. **Instrument Miniaturization**: The mass of an instrument represents one of the most critical constraints in the design of space missions.^213^ With each gram of scientific gear, the available propellant shrinks or the types of instruments that can be utilized are curtailed.^172,218^ Consequently, meticulous attention is required in the development of compact spectrophotometers like the IceShIP platform, which represents a first-of-its-kind approach to deploy state-of-the-art analytical instrumentation in small volume, mass, and power consumption envelopes, specifically designed to survive the extreme accelerations (high-g loads) experienced during impact events.^172^ Exemplified by compact Raman and LIBS payloads (e.g., SuperCam, MarSCoDe) and the development of integrated platforms such as IceShIP, this approach enables high-performance analysis within tight resource envelopes.^219^
ii. **Rigorous Qualification Protocols**: All newly developed space instrumentation must undergo rigorous qualification processes to be considered suitable for space deployment.^200,212^ Each element is obligated to meet the expectations laid out by the European Cooperation for Space Standardization (ECSS), specifying detailed testing strategies that include vibration, thermal cycling, electromagnetic compatibility, and radiation exposure evaluations.^220,221^ The malfunction of a single component can jeopardize a billion-euro mission, rendering qualification testing imperative. The Lunar Flashlight mission, launched in 2022 to retrieve new data on the distribution of water ice frost on Moon’s permanently shadowed regions (PSRs) using laser spectroscopy, concluded early because of a failure in its propulsion system.^222^
  Ground-based optical telescopes like IGGCAS-LOPS Planetary Atmosphere Spectroscopic Telescope (PAST) can be dedicated to discover probable planetary conditions to train planetary research equipment.^5^ Regular calibration of the instrument is also a critical concern as the readings are often affected by instrument response, environmental conditions, and time-dependent changes. Without regular calibration, it would be impossible to know whether changes in measured chemistry reflect real differences or simply instrument drift or altered performance. A method to do this calibration check could be carrying some calibration targets (CTs) with known, well-characterized compositions carried onboard by the rover. A study by Ytsma and co-workers showed that both Mars spectroscopic instruments ChemCam and SuperCam remained stable and reliable over time, since the CT did not degrade and calibration performance was reproducible.^223^ A trade-off was also revealed: Curiosity achieved strong accuracy but over a limited chemical range, while Perseverance sampled a wider range of compositions with slightly reduced accuracy, highlighting the importance of CT design for balancing realism and precision in planetary geochemical measurements. Spectral drift caused by environmental variations can significantly affect the accuracy of extraterrestrial LIBS measurements, making robust wavelength calibration essential for reliable qualitative and quantitative analyses. In a study, two correction strategies were developed to address different operational scenarios of the MarSCoDe LIBS system.^112^ The matching based on the global iterative registration (MGR) method was applied when the initial calibration remained valid, while a particle swarm optimization (PSO)-based approach was used when recalibration was required. Calibration and validation using multiple reference targets demonstrated an effective drift correction and improved spectral consistency.^112^
iii. **The Heritage Approach**: Even the most careful management of mass and resources is constrained by the realities of financial restrictions, which ultimately outlines the boundaries and goals of each mission. This challenge can be effectively mitigated through the heritage approach, which involves the utilization of previously deployed space equipment. A good example is the repurposing of the Mars Express spacecraft for Venus Express, alongside the incorporation of the spare VIRTIS instrument flight initially developed for the Rosetta mission.^220^ This instrument successfully mapped the southern hemisphere surface of Venus, despite not being specifically designed for this purpose. Utilizing this heritage approach brought about a significant decrease in mission costs by about 50%, concurrently helping to meet essential scientific targets.^220^ Arevalo et al. also applied this approach to obtain a laser of 266 nm by utilizing the Cr:Nd:YAG oscillator from the previously flown Lunar Orbiter Laser Altimeter (LOLA), which resulted in the laser energy being more than three times the maximum laser energy of the Mars Organic Molecule Analyzer (MOMA) instrument onboard the ExoMars rover.^224^ Reusing or adapting previously flown instruments significantly reduces development costs and risks, as demonstrated by missions leveraging legacy spectrometers and spacecraft designs.
iv. **Advancement in Instrumentation**: Difficulties in spectrometer instrumentation, such as spatial limitations, diminished light transmission efficiency, restricted spectral ranges, comparatively low resolutions for compact devices, and heightened sensitivity to misalignment, are also present.^26,225^ Kelly and co-workers reported a spatial heterodyne Raman spectrometer (SHRS), which is a fixed-grating interferometer, to address these problems.^26^ Nanosecond lasers, such as 193 nm excimer and 213 nm Nd:YAG lasers, are suitable to work with partially transparent geomaterials for their ablation effectiveness, hence for attachment with rovers.^226^ Femtosecond lasers, on the other hand, are suitable for in-laboratory uses, i.e., sample-return missions, due to their stoichiometric ablation without thermal effects, resulting in smoother craters and lower plasma temperatures and densities. Spectrometers, especially those synchronized with CCD or CMOS detectors, must cover a broad wavelength range with sufficient resolution to capture LIBS emissions effectively on space missions, despite introducing background noise.^226^ For enhanced sensitivity and resolution, echelle spectrometers coupled with intensified detectors are favored, although they present challenges such as increased costs and vulnerability to environmental factors.^226^
  Current missions (SuperCam on Perseverance, ChemCam on Curiosity) use nanosecond lasers, but understanding femtosecond plasma behavior could enable higher spatial resolution analysis, reduced matrix effects, and improved detection limits for trace elements, as evidenced by the study by Harilal et al. where fluorescence experiments demonstrated lower temperatures, higher ground-state populations, and early molecular formation for femtosecond plasmas and a depleted ground states due to hotter conditions with delayed fluorescence appearance for nanosecond plasmas.^227^
  A novel methodology for evaluating plasma-induced luminescence (PIL), which is the luminescent emissions elicited within a sample by the plasma induced by laser, in Martian environment was validated by Clavé and team.^209^ A scholarly investigation has accomplished a calibration-free laser-induced breakdown spectroscopy (CF-LIBS) method through the integration of baseline estimation and denoising, which facilitates nonlinear background subtraction and efficient peak fitting.^228^ Isotopic analysis through LIBS represents an advanced futuristic capability with implications for understanding planetary water sources and evolution. While challenging due to small isotopic shifts in emission lines, high-resolution LIBS systems can potentially detect D/H ratios, providing constraints on water origin and exchange with the atmosphere. The development of isotope-sensitive LIBS methods is an active area of research for future planetary missions.
v. **Terrestrial Analog Database Development**: A newly established repository of rock specimens and spectral data called the Planetary Terrestrial Analogue Library (PTAL) has been made public since 2021.^108^ This collection contains spectral measurements of Raman spectroscopy and LIBS, which have been verified through X-ray diffraction (XRD) and microscopic investigation. PTAL comprises a diverse range of natural rock specimens rather than purely minerals, thus providing a more comprehensive understanding of mineralogical and geochemical processes.^108^ To have a wide variety in the database of the terrestrial analogs (hot and cold deserts), studies at John Day Formation in Oregon (USA), Dry Valleys in Antarctica, Otago Formation (New Zealand), Jaroso Ravine, and Rio Tinto (Spain) have been conducted but the concept of employing terrestrial analogs is inherently complex with its own challenges.^108,229^ 50 years of planetary exploration has discovered giant shield volcanoes, solidified lava flows, extensive ash deposits, and volcanic vents on the rocky planets (Mercury, Venus, the moon, and Mars) and in the outer solar system (the moons of Jupiter and Saturn, and the larger asteroids).^230^ Terrestrial volcanic analogs not only act as sampling sites for studying the geological evolution of other planetary bodies, but also act as testing sites for equipment and training ground for crewed and uncrewed missions.^230^ Studying terrestrial analogs at the two poles of the earth can help in refining methods for detecting biosignatures. This includes defining baseline signals from abiotic processes, assessing how biosignatures are preserved over time, and identifying the chemical, physical, and isotopic markers produced by life in settings analogous to icy moons.^231^ Increased sample studies from these analog studies can then also be applied to train machine learning-based elemental quantification pipelines for LIBS in particular which is described in the next subsection.^211^ Moreover, various spectral databases like NIST, IRUG, SOLSA, RRUFF, etc. are openly available for Raman spectroscopy and LIBS spectra comparison and analysis for a large pool of target materials ranging from biomolecules to minerals.^232–235^

  The data derived from the terrestrial analogs cannot represent a flawless geochemical correspondence to the crusts of other planets, owing to the disparities between Earth’s atmosphere and those of other celestial bodies. The information obtained from the spectra of planetary analog sites helps reveal the geochemical history of Earth. By comparing these data with observations from other planetary bodies, scientists can better understand and refine theories about a planet’s formation. These studies are driven by humanity’s curiosity about the origins of the universe, but our understanding remains limited to hypotheses based on data collected during planetary exploration missions. Care must always be taken when drawing conclusions about geological or biological formation processes, since every celestial body has its own unique atmosphere that facilitates mineral or biomolecule creation, and the presence or absence of certain molecules/moieties in different ratios can lead to the development of different evolutionary story.
vi. **Advanced Chemometric Methods**: The exploration of planetary bodies has necessitated the development of advanced analytical techniques to accurately characterize their mineral compositions. Due to recent advancement in the computational science and analytics, there has been a surge of publications which have advanced the field of analytical techniques in the planetary exploration area.^76,193,236–238^

  Multivariate models using partial least-squares (PLS) and least absolute shrinkage and selection (LASSO) algorithms are generally used to predict the abundance of major elements based on LIBS spectra.^239^ In a study, Qi et al. introduced a new Raman quantitative model to distinguish various components from a mixture of minerals with accuracy in mixing ratios. The model was based on the linear relationships between Raman integrated intensities and mineral proportions and was independent of instrumental setup. Demaret and colleagues developed a Raman-based quantitative approach to determine organo-mineral compositions relevant to planetary biosignature searches.^35^ Simple models were validated on both benchtop and ExoMars RLS-like instruments, accounting for sample heterogeneity.^35^
  Additionally, a large section of researchers is trying to advance this domain through machine learning and neural networks. The use of an end-to-end ensemble convolutional neural network (ECNN) framework to directly model quantitative relationships between raw LIBS spectra and elemental composition, outperformed traditional linear and nonlinear methods in prediction accuracy and robustness while retaining interpretability, making it a promising tool for future planetary LIBS data analysis.^240^ Unlike controlled laboratory conditions, the working distance for an in situ LIBS instrument during planetary exploration exhibits natural variability. The significant spectral variations introduced by these differing distances present a substantial obstacle for the development and validation of chemometric models. Yang et al. demonstrated that traditional methods of spectral data processing could be effectively replaced by a chemometric approach utilizing a deep convolutional neural network (CNN) for the purpose of distance adjustment.^241^ Over 18,000 LIBS spectra gathered using a duplicate of the MarSCoDe instrument deployed in China’s Tianwen-1 Mars mission, was used for the purpose of this investigation.^241^ Discriminating minerals with close chemical compositions is another obstacle commonly encountered in LIBS analysis which can be mitigated by the implementation of CNN as mentioned by this team in another article.^242^ Berlanga et al. also used CNN to analyze low-intensity Raman spectra of micas, amphiboles, and mixed minerals and was able to distinguish them with +99% success.^243^
  Quantitative elemental analysis using LIBS in planetary exploration is strongly affected by laser energy fluctuations, matrix effects, and instrumental noise, leading to a limited prediction accuracy for unknown targets. To address these challenges, Wan and co-workers proposed an adaptive quantitative analysis approach for the MarSCoDe LIBS instrument based on support vector regression optimized using a particle swarm optimization algorithm. The model was trained on a large laboratory data set acquired under simulated Martian conditions and validated through comparative analysis and onboard calibration measurements, demonstrating improved accuracy, robustness, and suitability for in situ Martian material analysis.^244^
  Hence, the integration of advanced quantitative analysis techniques, such as multivariate models, machine learning, and neural networks, significantly enhances the efficacy of mineral composition analysis through LIBS and Raman spectroscopy, particularly by improving prediction accuracy and robustness in variable extraterrestrial environments, thereby facilitating superior material identification and contributing to a broader understanding of planetary compositions in the context of potential biosignature discovery.
vii. **A Multitechnique Strategy**: So far, the studies have concluded that Raman spectroscopy is the best technique applicable toward mineral studies.^38^ The established use of Raman combined with LIBS through the SuperCam on the Perseverance Mars rover for geochemical detection needs to be further extended toward other applications.^148,226^ At the same time, it is essential to pair it up with PL for a more accurate analysis of regolith. Works have been reported that bring emission and reflectance spectroscopies with Raman spectroscopy,^38^ but an established working triad of Raman–LIBS–PL still is missing for remote sensing in space applications. In 2021, Dhanada et al. mentioned in detail various advantages for using this triad of Raman–LIBS–PL particularly for multimodal spectroscopic applications, which extended from archeology, mineralogy, planetary explorations, clinical chemistry, and biomedical industry to environmental monitoring.^56^
  LIBS is predominantly advantageous for elemental analysis, whereas fluorescence and Raman spectroscopies are more proficient in molecular characterization. The amalgamation of these techniques facilitates the retrieval of both elemental and molecular data from a singular sample, which is exceedingly beneficial for routine applications.^56^ Nonetheless, actual samples frequently exhibit intricate spectral patterns attributable to their complex nature, intercomponent interactions, and suboptimal experimental conditions. For example, strong fluorescent backgrounds can obstruct Raman analysis, while LIBS may fail to yield significant molecular-composition information. Multimodal spectroscopy serves to mitigate these challenges by amalgamating diverse techniques. The confluence of these methodologies enables the creation of extensive data sets, which can be analyzed through sophisticated data acquisition methods, cloud computing, artificial intelligence (AI), and - learning (ML) algorithms for objective evaluation and predictive modeling.^56^
  A first study of its kind by Veneranda and team demonstrated that combining spectroscopic information from Raman, LIBS, and visible–infrared (VIS–IR) analyses significantly improves the discrimination of carbonate minerals relevant to SuperCam observations at Jezero crater.^102^ While single-technique approaches show specific limitations, midlevel data combination strategies effectively balance the contribution of each spectroscopy and eliminate biases caused by impurities or weak signals, achieving optimal classification accuracy.^102^ These results highlight the value of integrated spectroscopic and multivariate approaches for robust mineral identification in planetary exploration and future Mars sample selection.
  Hence, optical spectroscopy-based planetary exploration faces interconnected challenges spanning instrumentation, environment, cost, and data interpretation. These are increasingly mitigated through miniaturization, heritage utilization, terrestrial analogue studies, advanced instrumentation, rigorous testing, and multitechnique integration supported by advanced data analytics. The implementation of Raman–LIBS–PL would be a step toward that direction which can further be combined with multimodal machine learning models developed to identify targets based on a multimodal fusion architecture which already has been reported to be possible.^245^ A thorough comprehension of celestial entities is fundamentally dependent on high-resolution orbital remote sensing, direct exploration, and the acquisition of physical specimens.^246^

## 8 Conclusions

Laser-based optical spectroscopic techniques of Raman spectroscopy, laser-induced breakdown spectroscopy, and photoluminescence spectroscopy have matured into indispensable tools for planetary exploration, enabling comprehensive in situ characterization of extraterrestrial materials across diverse environments from the Martian surface to the icy satellites of the outer solar system through landmark deployments of SuperCam on the Perseverance rover, SHERLOC instrument, and Chandrayaan-3 LE-LIBS instrument. The past five years (2021–2025) have witnessed remarkable advances in instrument capabilities, operational strategies, and scientific discoveries that establish laser spectroscopy as a cornerstone technology for current and future planetary missions such as the Artemis II program and Exomars 2028. Terrestrial analogue research has proven to be essential for validating instruments and operational strategies on extraterrestrial bodies. These analogue studies have informed mission operations, spectral library development, and data interpretation approaches that directly benefit planetary missions.

Explicit comparisons among Raman, LIBS, and PL under planetary conditions reveal complementary strengths: Raman excels at molecular fingerprinting and mineral identification, LIBS provides rapid elemental composition analysis, and PL detects trace luminescent species and organic fluorophores. No single technique or wavelength optimizes all applications; strategic integration of multiple techniques (LIBS + Raman, Raman + PL) and time-resolved methods (time-gating, pulsed excitation, ultrafast spectroscopy) maximizes scientific return. In addition, future instrument design should link wavelength choice to mission objectives: deep-UV for astrobiology-focused missions (Europa, Enceladus, Mars subsurface), visible Raman for general mineralogy and hydration studies, and time-resolved experiments to overcome fluorescence when wavelength selection alone is insufficient. Future missions will also increasingly employ onboard machine learning for adaptive measurement strategies that optimize the scientific return.

The collaborative utilization of Raman, LIBS, and PL spectroscopies within hybrid systems can establish a robust, adaptable, and comprehensive analytical framework. This integration can enable complementary elemental and molecular insights, reduce ambiguity, and enhance the detection of hydration, organics, and biosignatures. This multitechnique approach would not only accelerate the pace of discovery but also foster collaboration among researchers, paving the way for groundbreaking advancements in planetary science. As we expand human and robotic exploration to the moon, Mars, and beyond, laser-based optical spectroscopy will continue to provide the analytical foundation for understanding planetary processes, assessing habitability, detecting biosignatures, and enabling sustainable exploration. Fully integrated Raman–LIBS–PL systems employing shared optics and spectrometers can demonstrate the feasibility of multimodal analysis in compact form factors suitable for small rovers, landers, and aerial platforms.

This Review serves as a comprehensive reference for physicists, geochemists, planetary scientists, and instrument developers, enabling them to compare the suitability of different optical spectroscopic techniques among Raman spectroscopy, LIBS, and photoluminescence spectroscopy for specific planetary applications, identify knowledge gaps and technological limitations that require further research, and guide future developments for remote detection and in situ characterization of extraterrestrial environments.
